# CXCR4-targeted nanotoxins induce GSDME-dependent pyroptosis in head and neck squamous cell carcinoma

**DOI:** 10.1186/s13046-022-02267-8

**Published:** 2022-02-04

**Authors:** Elisa Rioja-Blanco, Irene Arroyo-Solera, Patricia Álamo, Isolda Casanova, Alberto Gallardo, Ugutz Unzueta, Naroa Serna, Laura Sánchez-García, Miquel Quer, Antonio Villaverde, Esther Vázquez, Xavier León, Lorena Alba-Castellón, Ramon Mangues

**Affiliations:** 1 Institut d’Investigació Biomèdica Sant Pau (IIB-Sant Pau), Sant Quintí, 77, 08041 Barcelona, Spain; 2 Institut de Recerca contra la Leucèmia Josep Carreras, 08025 Barcelona, Spain; 3 CIBER de Bioingeniería, Biomateriales y Nanomedicina (CIBER-BBN), Monforte de Lemos 3-5, 28029 Madrid, Spain; 4 Department of Pathology, Hospital de la Santa Creu i Sant Pau, Sant Quintí, 89, 08041 Barcelona, Spain; 5 Institut de Biotecnologia i de Biomedicina, Universitat Autònoma de Barcelona, 08193 Bellaterra, Spain; 6 Departament de Genètica i de Microbiologia, Universitat Autònoma de Barcelona, 08193 Bellaterra, Spain; 7 Department of Otorhinolaryngology, Hospital de la Santa Creu i Sant Pau, Universitat Autònoma de Barcelona, Sant Quintí, 89, 08041 Barcelona, Spain; 8 Department of Surgery, Hospital de la Santa Creu i Sant Pau, Universitat Autònoma de Barcelona, Sant Quintí, 89, 08041 Barcelona, Spain; 9 Institut de Biotecnologia i de Biomedicina and Departament de Genètica i de Microbiologia, Universitat Autònoma de Barcelona and CIBER, Bellaterra, Barcelona, Spain; 10 Institut d’Investigacions Biomèdiques Sant Pau, Hospital de Sant Pau, CIBER and Josep Carreras Research Institute, 08041 Barcelona, Spain; 11 Institut d’Investigacions Biomèdiques Sant Pau, Hospital de Sant Pau and Josep Carreras Research Institute, 08041 Barcelona, Spain

**Keywords:** Targeted drug delivery, CXCR4, HNSCC, Pyroptosis, GSDME

## Abstract

**Background:**

Therapy resistance, which leads to the development of loco-regional relapses and distant metastases after treatment, constitutes one of the major problems that head and neck squamous cell carcinoma (HNSCC) patients currently face. Thus, novel therapeutic strategies are urgently needed. Targeted drug delivery to the chemokine receptor 4 (CXCR4) represents a promising approach for HNSCC management. In this context, we have developed the self-assembling protein nanotoxins T22-PE24-H6 and T22-DITOX-H6, which incorporate the de-immunized catalytic domain of *Pseudomonas aeruginosa* (PE24) exotoxin A and the diphtheria exotoxin (DITOX) domain, respectively. Both nanotoxins contain the T22 peptide ligand to specifically target CXCR4-overexpressing HNSCC cells. In this study, we evaluate the potential use of T22-PE24-H6 and T22-DITOX-H6 nanotoxins for the treatment of HNSCC.

**Methods:**

T22-PE24-H6 and T22-DITOX-H6 CXCR4-dependent cytotoxic effect was evaluated in vitro in two different HNSCC cell lines. Both nanotoxins cell death mechanisms were assessed in HNSCC cell lines by phase-contrast microscopy, AnnexinV/ propidium iodide (PI) staining, lactate dehydrogenase (LDH) release assays, and western blotting. Nanotoxins antitumor effect in vivo was studied in a CXCR4^+^ HNSCC subcutaneous mouse model. Immunohistochemistry, histopathology, and toxicity analyses were used to evaluate both nanotoxins antitumor effect and possible treatment toxicity. GSMDE and CXCR4 expression in HNSCC patient tumor samples was also assessed by immunohistochemical staining.

**Results:**

First, we found that both nanotoxins exhibit a potent CXCR4-dependent cytotoxic effect in vitro. Importantly, nanotoxin treatment triggered caspase-3/Gasdermin E (GSDME)-mediated pyroptosis. The activation of this alternative cell death pathway that differs from traditional apoptosis, becomes a promising strategy to bypass therapy resistance. In addition, T22-PE24-H6 and T22-DITOX-H6 displayed a potent antitumor effect in the absence of systemic toxicity in a CXCR4^+^ subcutaneous HNSCC mouse model. Lastly, GSDME was found to be overexpressed in tumor tissue from HNSCC patients, highlighting the relevance of this strategy.

**Conclusions:**

Altogether, our results show that T22-PE24-H6 and T22-DITOX-H6 represent a promising therapy for HNSCC patients. Remarkably, this is the first study showing that both nanotoxins are capable of activating caspase-3/GSDME-dependent pyroptosis, opening a novel avenue for HNSCC treatment.

**Supplementary Information:**

The online version contains supplementary material available at 10.1186/s13046-022-02267-8.

## Background

One of the main problems of head and neck squamous cell carcinoma (HNSCC) therapy is that up to 60% of patients develop loco-regional relapses and up to 30% distant metastases after treatment, dramatically affecting their survival. Currently, these HNSCC patients are no longer candidates for a curative therapy and the main goals are palliation and prolongation of patient survival [1, 2]. Thus, the development of drug resistance, which leads to treatment failure and relapses, constitutes a major issue in current HNSCC treatment, highlighting the urge for novel therapeutic strategies [3–6].

In this context, targeting the chemokine receptor 4 (CXCR4) has emerged as a promising approach in cancer treatment. CXCR4 overexpression in HNSCC primary tumors associates with a higher risk of developing loco-regional recurrences and distant metastases after treatment and worse overall survival [7, 8]. Moreover, preclinical and clinical data suggest that this chemokine pathway contributes to a resistant phenotype [8]. Thus, targeted drug delivery to CXCR4-overexpressing cells represents a promising antitumor strategy in HNSCC treatment.

In the last years, different protein toxins have gained relevance as moieties of antitumor drugs because of their interesting properties that can be exploited in clinical oncology [9–11]. Toxins display a great cytotoxicity in a wide range of cancer cells, presenting mechanisms of action capable of killing not only proliferating, but also quiescent cells [10]. In addition, toxins can be recombinantly produced enabling large scale production and purification. All these facts make them ideal candidates to replace current chemotherapeutic drugs. However, to prevent undesired off-target toxicities, targeted drug delivery specifically to tumor cells is key for the translation of toxin-based drugs to the clinic. In this context, different antibody-drug conjugates (ADCs) and immunotoxins have exploited this strategy. An example is the immunotoxin denileukin diftitox, that incorporates the interleukin-2 (IL-2) fused to the diphtheria exotoxin domain to target T cell lymphoma cells that overexpress the IL-2 receptor. However, it was withdrawn from the market in 2014 due to life-threatening toxicity in patients [12, 13]. In fact, immunotoxin translation to the clinic has been jeopardized by severe off-target toxicities, especially because of their high immunogenicity and reduced targeting capacity, which limits their long-term use in patients [12, 13]. On the other hand, different ADCs have also been tested for the treatment of HNSCC, such as ABBV-321, an EGFR-targeting antibody conjugated to a pyrrolobenzodiazepine (PBD) dimer cytotoxic molecule [14]. Nevertheless, ADCs present important drug leakage during circulation, also limiting their clinical use [15–17]. Altogether, ADCs and immunotoxins present severe off-target toxicities, which dramatically narrow down their therapeutic window.

In this context, we have developed self-assembling protein nanoparticles which incorporate the de-immunized catalytic domain of *Pseudomonas aeruginosa* (PE24) exotoxin A or the diphtheria exotoxin (DITOX) domain from *Corynebacterium diphtheriae*, that specifically target CXCR4-overexpressing (CXCR4^+^) cancer cells through the T22 peptide ligand. These nanotoxins, named T22-PE24-H6 and T22-DITOX-H6 respectively, are recombinantly produced in *Escherichia coli*, where they self-assemble into multimeric nanoparticles. This fact enables a greater payload capacity while increasing the number of ligands per nanoparticle, which endows superselectivity [18]. Moreover, both nanotoxins are produced in a single step process, avoiding chemical conjugation steps, which allows a straightforward production and purification while preventing drug leakage [19].

Here, we describe the cytotoxic and antitumor effect of these two novel nanotoxins, T22-PE24-H6 and T22-DITOX-H6 that specifically target CXCR4^+^ cells, for the treatment of HNSCC. First, we evaluate the CXCR4-dependent cytotoxic effect of both nanotoxins in two different HNSCC cell lines. Moreover, we analyze the mechanisms of cell death induced by both T22-PE24-H6 and T22-DITOX-H6, finding that they are capable of activating caspase-3/Gasdermin E (GSDME) mediated pyroptosis. Since the activation of anti-apoptotic pathways is a main mechanism of resistance to both chemotherapy and radiotherapy in HNSCC [3, 5, 6, 20], the development of drugs capable of triggering cell death pathways alternative to apoptosis is an important avenue of research that could increase cure rate in HNSCC patients. Importantly, T22-PE24-H6 and T22-DITOX-H6 also present a potent antitumor effect in the absence of systemic toxicity in a CXCR4^+^ subcutaneous HNSCC mouse model. Lastly, we incorporate clinical data showing that GSDME (also named DFNA5) is overexpressed in tumor tissue of HNSCC patients, highlighting the relevance of this strategy. Thus, activating caspase-3/GSDME-dependent pyroptosis specifically in CXCR4^+^ HNSCC tumor cells may represent a novel and enticing approach for the treatment of HNSCC patients. Remarkably, this is the first study showing that T22-PE24-H6 and T22-DITOX-H6 activate caspase-3/GSDME mediated pyroptosis in oncotherapy.

## Methods

### Nanoparticles production, purification, and characterization

T22-PE24-H6 and T22-DITOX-H6 production, purification, and characterization have been previously described [19]. T22-PE24-H6 nanoparticles self-assemble into ~ 60 nm nanoparticles, whereas T22-DITOX-H6 form 38 and 90 nm nanoparticles.

### Cell lines and cell culture

UM-SCC-22A (22A) and UM-SCC-74B (74B) human papillomavirus negative (HPV^−^) HNSCC cell lines [21] were kindly provided by Dr. R. H. Brakenhoff and Dr. Gregory Oakley respectively. 22A mock, 74B mock, 22A-CXCR4^+^, and 74B-CXCR4^+^ were obtained by lentiviral transduction with the plasmids pLenti-III-UbC-luc and pLenti-III-UbC-CXCR4-2A-luc (abm, Vancouver, Canada) respectively, as already described in previous work [22]. HNSCC cell lines were cultured in Dulbecco’s Modified Eagle’s Medium (DMEM) (Gibco, Life Technologies) supplemented with 10% Fetal Bovine Serum (FBS), 100 U/mL penicillin/ streptomycin, and 2 mM glutamine (Life Technologies) and incubated at 37 °C and 5% CO_2_ in a humidified atmosphere. CXCR4 expression in 22A mock, 74B mock, 22A-CXCR4^+^, and 74B-CXCR4^+^ has been already evaluated in previous work [22].

575 and 909 patient-derived cell cultures were obtained from two HNSCC patient tumor samples with high CXCR4 tumor expression. Tumor samples were disaggregated by incubation in Trypsin-EDTA 0.25% (Gibco, Life Technologies) for 2 h at 4 °C, followed by further incubation with collagenase type II (200 mg/mL, Life Technologies) and DNase (20 mg/mL, Sigma-Aldrich) for 2 h at 37 °C. After some mechanical disruption, samples were filtered through a 40 μm mesh, and cultured. Epithelial cell enrichment was performed by differential trypsinization and maintaining the cells in Defined Keratinocyte-SFM medium (Gibco, Life Technologies). 575 and 909 cell cultures were maintained Dulbecco’s Modified Eagle Medium: Nutrient Mixture F-12 (DMEM/F-12) (Gibco, Life Technologies) supplemented with 10% Fetal Bovine Serum (FBS), 100 U/mL penicillin/ streptomycin, 2 mM glutamine, and 400 ng/ml hydrocortisone (Life Technologies) at 37 °C and 5% CO_2_ in a humidified atmosphere.

### Patient samples

HNSCC patient samples were obtained by the Otorhinolaryngology Department of the Hospital de la Santa Creu i Sant Pau (Barcelona, Spain) in accordance with the Institutional Review Board of the institution. Written informed consent was acquired from all the patients involved in this study.

### Cell viability assays

Cell viability upon T22-PE24-H6 and T22-DITOX-H6 exposure was assessed with the Cell Proliferation Kit II (XTT) (Roche) according to the manufacturer’s instructions. Cells were seeded in 96-well plates (5000 cells/well for 22A and 2500 cells/well for 74B) and treated with either buffer (166 mM NaCO_3_H pH 8) or different concentrations of T22-PE24-H6 or T22-DITOX-H6 (0–50 nM) for 48 h. For AMD3100 blocking experiments, 1 μM AMD3100 was added 1 h prior to the addition of the nanotoxins. For the zVAD pre-treatment, 100 μM zVAD (Calbiochem) was added to the plates and incubated at 37 °C for 2 h before nanotoxin treatment. After 48 h, XTT reagent was added to the plate and further incubated at 37 °C for 4 h, then absorbance, which directly correlates to the number of viable cells, was measured using a multi-well spectrophotometer (FLUOstar Optima, BMG Labtech). All experiments were performed in triplicate.

### Flow cytometry

Cell death induced by T22-PE24-H6 and T22-DITOX-H6 was further assessed using the Annexin V-FITC / propidium iodide (PI) detection kit (Merck Millipore) following manufacturer’s instructions. 74B-CXCR4^+^ cells were cultured in 6-well plates (25,000 cells/well) and exposed to 50 nM of either of the two nanotoxins for different times (15 h, 24 h, and 48 h). Cells were analyzed by MACSQuant analyzer flow cytometry with the MACS Quantify version 2.3 software (Miltenyi Biotech). The experiment was performed in triplicate.

### LDH release assay

LDH release from 22A-CXCR4^+^ and 74B-CXCR4^+^ cells upon nanotoxin exposure was studied using the CytoTox 96 Non-Radioactive Cytotoxicity Assay (Promega). Cells were seeded in 96-well plates (5000 cells/well for 22A and 2500 cells/well for 74B) and exposed to the nanotoxins (5 nM for T22-PE24-H6 in 22A-CXCR4^+^ cell line, 50 nM in the rest of conditions). zVAD (Calbiochem) pre-treatment was performed at a 100 μM concentration and incubated at 37 °C for 2 h prior to nanotoxin addition. After 48 h of treatment, cytotoxicity assay reagents were added according to the manufacturer’s instructions, and the absorbance at 492 nm was measured using a multi-well spectrophotometer (FLUOstar Optima, BMG Labtech). All experiments were performed in triplicate.

### Western blotting

T22-PE24-H6 and T22-DITOX-H6 cell death mechanisms were further studied by western blotting (WB). For that, 22A-CXCR4^+^ and 74B-CXCR4^+^ cells were treated with either of the nanotoxins (5 nM for T22-PE24-H6 in 22A-CXCR4^+^ cell line, 50 nM in the rest of conditions) for different times (15 h, 24 h, and 48 h). In the zVAD conditions, the pan-caspase inhibitor (Calbiochem) was added at 100 μM and incubated for 2 h before nanotoxin treatment. WB was also used to evaluate the GSDME expression in two patient-derived cultures (575 and 909). Cells were lysed in RIPA buffer (Sigma) containing proteinase inhibitors (Roche) and phosphatase inhibitors (Roche). The protein extracts (50 μg) were subjected to SDS-PAGE and transferred to a nitrocellulose blotting membrane (GE Healthcare life sciences). After blockage with 5% skim milk in TBS-T for 1 h at room temperature, membranes were incubated overnight at 4 °C with the primary antibodies: anti-human caspase-3 (1:1000, BD Biosciences), cleaved caspase-3 (1:1000, Cell Signaling), PARP (1:2000, BD Biosciences), GSDME (1:1000, abm), and α/β-tubulin (1:1000, Cell Signaling). After washing with TBS-T to remove nonspecific antibody binding, membranes were incubated with the corresponding secondary antibodies (1:10,000, Jackson Immune Research) for 1 h at room temperature. Finally, membranes were further washed with TBS-T and visualized with the SuperSignal™ West Pico Chemiluminescent Substrate and SuperSignal™ West Femto Maximum Sensitivity Substrate (Thermo Scientific) using the ChemiDoc XRS+ imaging system (Biorad). Pro-caspase-3, cleaved caspase-3, cleaved PARP, and cleaved GSDME levels were quantified using Fiji, ImageJ software. The densitometric analysis was performed by dividing the value of each nanotoxin-treated sample by the buffer-treated sample and normalized by the loading control (α/β-tubulin). All experiments were performed at least in triplicate.

### In vivo experiments

Four-week-old female Swiss nude mice (NU (Ico)-*Foxn1*^*nu*^) weighing 18–25 g were purchased from Charles River (France). Animals were housed in a specific pathogen-free (SPF) environment with sterile food and water ad libitum. All animal experiments were approved by the Hospital de la Santa Creu i Sant Pau Animal Ethics Committee.

The subcutaneous tumor model was generated by subcutaneous injection of 10 million 74B-CXCR4^+^ cells in both flanks of the animal. To assess T22-PE24-H6 and T22-DITOX-H6 antitumor effect, animals bearing tumors around 60–100 mm^3^ were randomized into three groups (*n* = 10 per group). Animals were intravenously administered buffer (166 mM NaCO_3_H pH 8), or 10 μg of either T22-PE24-H6 or T22-DITOX-H6 every day up to 8 doses. Animal body weight and tumor size were measured with a caliper (tumor volume = width^2^ x length/2) through the time course of the treatment. Animals were euthanized 48 h after the last dose, when tumors were weighted and collected together with different organs for later analysis. Plasma was also obtained by centrifugation of total blood, extracted from the animals by intracardiac puncture.

To evaluate the possible long-term toxicity of the nanotoxins treatment, four-week-old female non-tumor bearing Swiss nude mice (NU (Ico)-*Foxn1*^*nu*^) were intravenously administered buffer or 10 μg of either T22-PE24-H6 or T22-DITOX-H6 daily up to 8 doses, similarly to the antitumor effect experiment. Animals were weighted twice a week during the study. After the last dose, blood samples were collected from the tail every week to assess cell blood count (CBC). Animals were euthanized 4 weeks after the end of the treatment and different organs were collected for histopathological analysis.

### Histopathology, DAPI staining, and immunohistochemical analysis

4 μm paraffin-embedded sections obtained from tumor patient samples collected at the Hospital de la Santa Creu i Sant Pau, as well as tumors and organs extracted from the animals were used to performed different histopathological analysis. Organ sections were stained with H&E and analyzed by two independent observers (one section of the whole organ/tumor). Cell death in tumor tissues was assessed by DAPI staining, paraffin-embedded sections were dewaxed, rehydrated, and permeabilized with 0.5% Triton X-100. Then, slides were stained with DAPI mounting medium (ProLong™ Gold Antifade Mountant, Thermo Scientific) and visualized by fluorescence microscopy. Representative pictures were taken using an Olympus DP73 digital camera and the number of dead cells was quantified by counting the number of condensed nuclei per 10 high-power fields (magnification 400x). Immunohistochemical (IHC) staining of animal tumors was performed to study the cytotoxic effect of the nanotoxin treatment. CXCR4 (1:200, Abcam. Retrieval pH high, Dako) and F4/80 (ready to use, Dako) IHC were performed in a DAKO Autostainer Link48 following the manufacturer’s instructions. Similarly, GSDME IHC staining (1:300, Abcam. Retrieval pH high, Dako) was assessed in patient tumor samples. Representative images were captured using an Olympus DP73 digital camera and processed with the Olympus CellD Imaging 3.3 software. CXCR4 and F4/80 expression levels in tumors were quantified as mean gray values using Fiji, ImageJ software.

### Toxicity analyses in plasma and total blood samples

To further evaluate the toxicity of T22-PE24-H6 and T22-DITOX-H6 treatment in mice, plasma glutamic oxaloacetic transaminase (GOT) and glutamic pyruvic transaminase (GPT) enzyme activities, as well as creatinine and uric acid levels were assessed in plasma samples using commercial kits (Roche) in a COBAS 6000 autoanalyzer (Roche).

Cell blood count (CBC) from buffer and nanotoxin-treated animals was analyzed using the Mindray BC-5000 Vet hematology analyzer.

### Statistical analysis

Data was represented as mean ± Standard error (SEM). Statistical analyses were performed using the GraphPad Prism 5 software (GraphPad Software, San Diego, California USA). Results were analyzed by Student t-test. Differences were considered statistically significant when *p*-values < 0.05.

## Results

### T22-PE24-H6 and T22-DITOX-H6 exhibit a potent CXCR4-dependent cytotoxicity in HNSCC cell lines

**Fig. 1:**
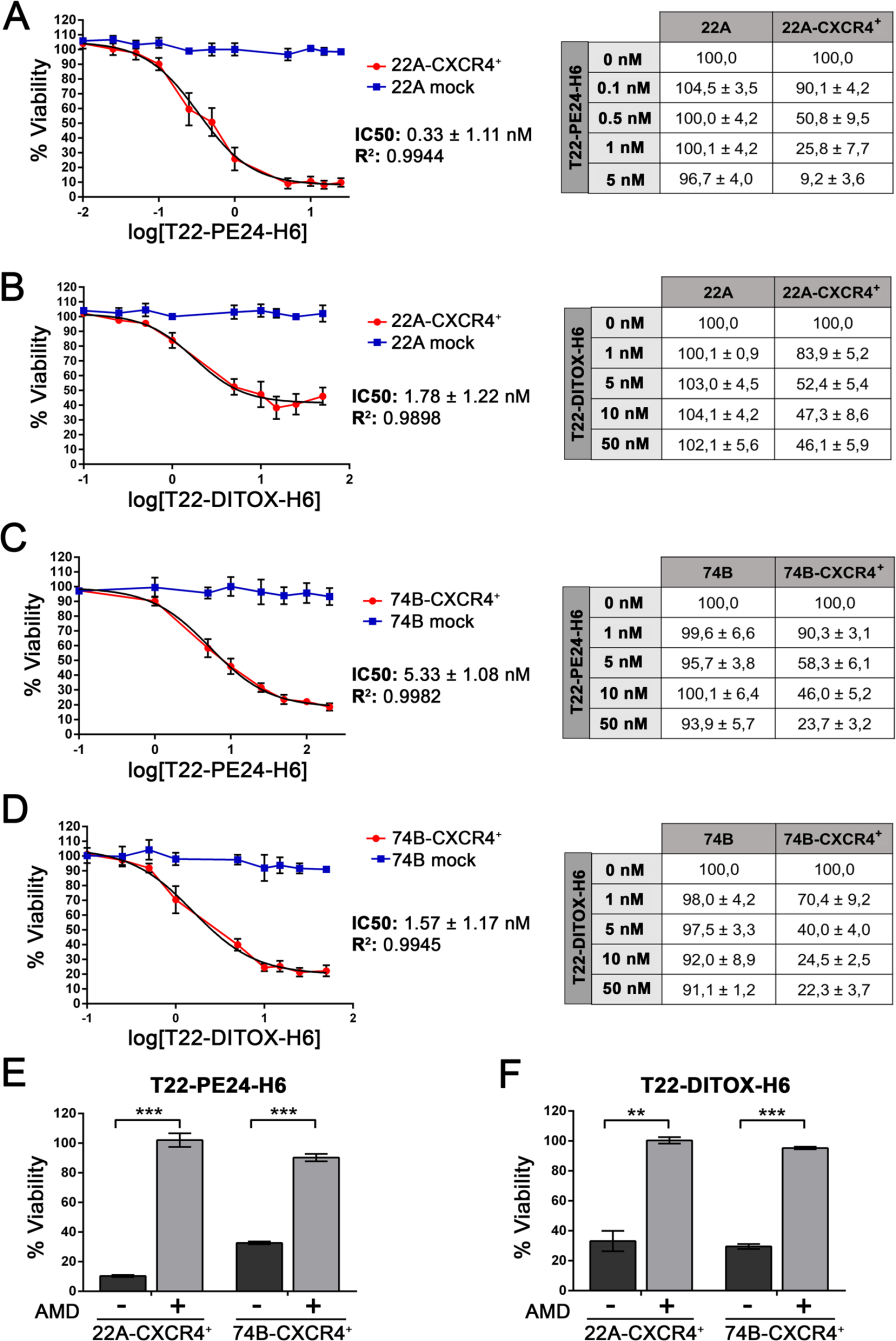
T22-PE24-H6 and T22-DITOX-H6 CXCR4-dependent cytotoxic effect in HNSCC cell lines. **A** and **B**) T22-PE24-H6 (**A**) and T22-DITOX-H6 (**B**) cytotoxic effect (0-50 nM) after 48 h of exposure in 22A mock and 22A-CXCR4^+^ cell lines represented as percentage of cell viability and IC_50_ values. **C** and **D**) T22-PE24-H6 (**C**) and T22-DITOX-H6 (**D**) cytotoxic effect (0–50 nM) after 48 h of exposure in 74B mock and 74B-CXCR4^+^ cell lines represented as percentage of cell viability and IC_50_ values. **E** and **F**) AMD3100 blocking assay (1 μM) in 22A-CXCR4^+^ and 74B-CXCR4^+^ cell lines treated with T22-PE24-H6 (**E**) (5 nM for 22A-CXCR4^+^ and 50 nM for 74B-CXCR4^+^) and T22-DITOX-H6 (**F**) (50 nM) for 48 h. ** *p* < 0.01; *** *p* < 0.001. Each column represents the mean value of three biological replicates. Statistical analysis performed by Student t-test. Error bars indicate SEM

T22-PE24-H6 and T22-DITOX-H6 cytotoxic effect was evaluated in vitro in two HNSCC cell lines, 22A mock and 22A-CXCR4^+^; and 74B mock and 74B-CXCR4^+^. Both 22A mock and 74B mock cell lines were negative for the receptor, whereas 22A-CXCR4^+^ and 74B-CXCR4^+^ displayed a strong CXCR4 membrane expression, as it has been already studied by flow cytometry and immunocytochemistry in previous work [22]. Cells were exposed to different concentrations of either of the two nanotoxins (0–50 nM) for 48 h before assessing their viability. Both T22-PE24-H6 and T22-DITOX-H6 were able to induce cell death in 22A-CXCR4^+^ and 74B-CXCR4^+^ cells, which express high levels of CXCR4 in their membranes (Fig. 1A-D). Remarkably, nanotoxins present a potent cytotoxic effect, as they were capable of inducing cell death at low concentrations (nM range) (Fig. 1A-D). IC_50_ values were calculated for both nanotoxins, ranging between 1 and 5 nM, further supporting T22-PE24-H6 and T22-DITOX-H6 potent cytotoxicity (Fig. 1A-D). On the other hand, neither 22A mock nor 74B mock (CXCR4 negative cells) cell viability was altered upon nanoparticle exposure, suggesting a CXCR4-dependent cytotoxic effect (Fig. 1A-D).

T22-PE24-H6 and T22-DITOX-H6 CXCR4-dependent cytotoxicity was further corroborated by pre-incubating both 22A-CXCR4^+^ and 74B-CXCR4^+^ cells with the CXCR4 antagonist AMD3100, 1 h prior to the addition of the nanotoxins. Treatment with AMD3100 blocked nanotoxin binding to CXCR4, leading to a practically complete remission of their cytotoxic effect in both CXCR4^+^ cell lines (Fig. 1E and F).

Thus, both T22-PE24-H6 and T22-DITOX-H6 display a potent CXCR4-dependent cytotoxic effect in vitro in both HNSCC cell lines.

### T22-PE24-H6 and T22-DITOX-H6 nanotoxins induce pyroptosis in HNSCC cell lines

**Fig. 2:**
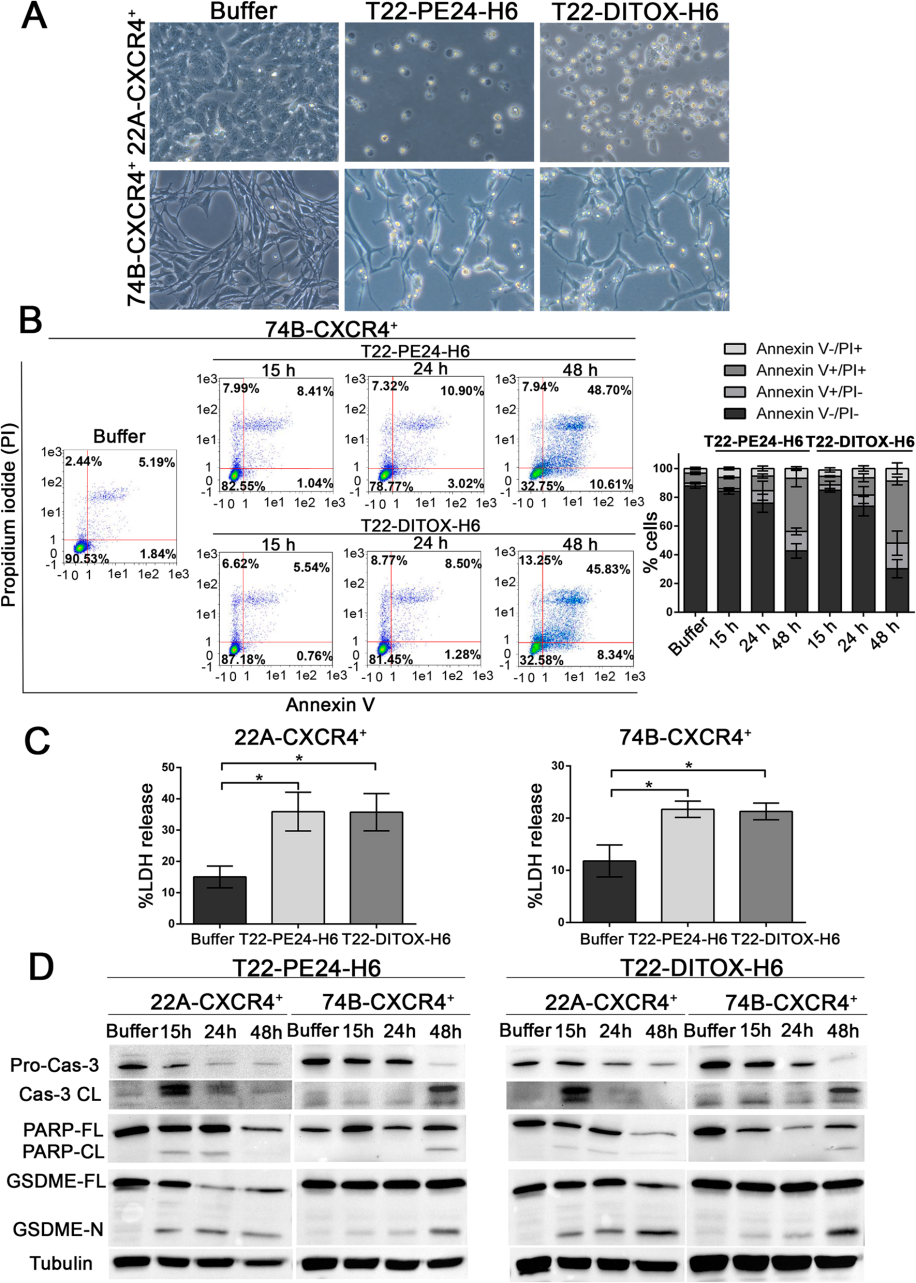
T22-PE24-H6 and T22-DITOX-H6 nanotoxins induce tumor cell pyroptosis. **A** Phase-contrast imaging of 22A-CXCR4^+^ and 74B-CXCR4^+^ cells treated with T22-PE24-H6 or T22-DITOX-H6 for 48 h exhibiting pyroptotic cell morphology (magnification 200x). **B** Flow cytometry analysis of 74B-CXCR4^+^ after 15 h, 24 h, and 48 h of exposure to T22-PE24-H6 or T22-DITOX-H6 stained with Annexin V-FITC and propidium iodide (PI). Percentage of stained cells is represented in the column graph. **C** LDH release from 22A-CXCR4^+^ and 74B-CXCR4^+^ exposed to either T22-PE24-H6 or T22-DITOX-H6 for 48 h. **D** Representative images of pro-caspase-3, cleaved caspase-3, PARP, GSDME, and tubulin immunoblotting in protein extracts from 22A-CXCR4^+^ and 74B-CXCR4^+^ cell lines treated with T22-PE24-H6 and T22-DITOX-H6 for 15 h, 24 h, and 48 h. * *p* < 0.05. Each column represents the mean value of three biological replicates. Statistical analysis performed by Student t-test. Error bars indicate SEM

The aforementioned potent CXCR4-dependent cytotoxic effect prompted us to investigate the mechanism of cell death induced by both T22-PE24-H6 and T22-DITOX-H6 nanotoxins. Interestingly, upon nanoparticle exposure, we observed a balloon-like morphology of the cells, which clearly differ from classic apoptotic blebbing (Fig. 2A). These swelling cells were especially noticeable in the 22A-CXCR4^+^ cultures treated with either of the two nanotoxins, although they could also be observed in the 74B-CXCR4^+^ cell line (Fig. 2A). Remarkably, this balloon-like shape is a characteristic of pyroptotic cell morphology.

In addition to these observations, we performed an Annexin V/ Propidium iodide (PI) assay by flow cytometry to further study the type of cell death induced by T22-PE24-H6 and T22-DITOX-H6. In classic apoptosis, cells first undergo an early apoptosis phase (Annexin V^+^/PI^−^), followed by a late apoptosis phase (Annexin V^+^/PI^+^), characterized by plasma membrane rupture and leakage. However, in this case we did not observe these phases, revealing a lytic type of cell death, as Annexin V/PI double-positive stained cells increased upon treatment with both nanotoxins (Fig. 2B).

To further elucidate the mechanism of cell death induced by both nanotoxins, we evaluated the LDH released from cells 48 h after nanoparticle treatment, which correlates with cell membrane disruption and leakage. In agreement with the previous findings, nanotoxin treated cells showed an increase in LDH release, further corroborating a lytic form of cell death (Fig. 2C).

Finally, we studied different cell death markers by western blotting to determine the exact mechanisms of cell death activated by T22-PE24-H6 and T22-DITOX-H6. We found an activation of caspase-3, PARP, and GSDME in both HNSCC cell lines treated with either of the two nanotoxins (Fig. 2D). 22A-CXCR4^+^, which has shown a greater sensitivity to both nanotoxins, presented cleaved caspase-3 at 15 h and 24 h, leading to the activation of PARP and GSDME also at 15 h and 24 h. On the other hand, activation of caspase-3, PARP, and GSDME in the 74B-CXCR4^+^ cell line was only observed at 48 h (Fig. 2D, Supplementary Fig. 1). In the last years, different studies have determined that pyroptosis, a lytic form of cell death, can be triggered by GSDME N-terminal domain which is activated by cleaved caspase-3, also responsible for the proteolytic activation of PARP during apoptosis [23, 24]. Interestingly, we observed the simultaneous activation of both PARP and GSDME upon nanotoxin treatment, which has also been described in other molecular therapies [25–28].

### T22-PE24-H6 and T22-DITOX-H6 activate caspase-3/GSDME-mediated pyroptosis in HNSCC cell lines

**Fig. 3:**
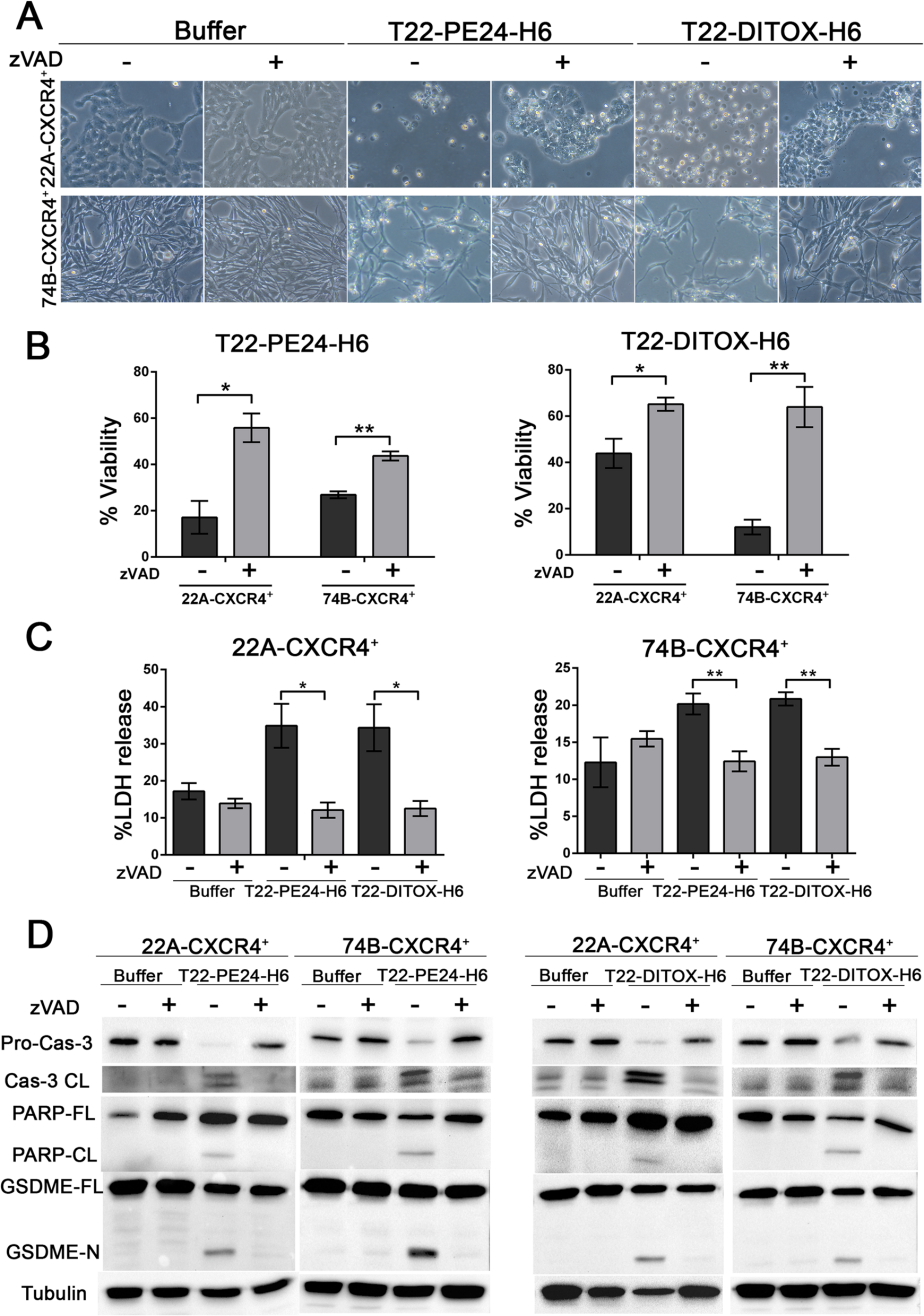
zVAD pre-treatment of 22A-CXCR4^+^ and 74B-CXCR4^+^ cells shows T22-PE24-H6 and T22-DITOX-H6 activation of caspase-3/GSDME-mediated pyroptosis. **A** Phase-contrast imaging of 22A-CXCR4^+^ and 74B-CXCR4^+^ cell lines with and without zVAD pre-treatment (100 μM) 1 h prior to the addition of T22-PE24-H6 or T22-DITOX-H6 (magnification 200x). zVAD clearly inhibits pyroptotic cell morphology in both cell lines. **B** Cell viability of 22A-CXCR4^+^ and 74B-CXCR4^+^ cells either pre-treated or not with zVAD before the addition of T22-PE24-H6 and T22-DITOX-H6 nanotoxins. **C** LDH release from 22A-CXCR4^+^ and 74B-CXCR4^+^ treated with T22-PE24-H6 or T22-DITOX-H6 for 48 h, with and without zVAD pre-treatment. **D** Representative images of pro-caspase-3, cleaved caspase-3, PARP, GSDME, and tubulin western blots of samples from 22A-CXCR4^+^ and 74B-CXCR4^+^ cell lines exposed to the inhibitor zVAD before nanotoxin treatment for 48 h. * *p* < 0.05; ** *p* < 0.01. Each column represents the mean value of three biological replicates. Statistical analysis performed by Student t-test. Error bars indicate SEM

In order to validate the previous findings which suggested that the caspase-3/GSDME pathway would be responsible for nanotoxin cytotoxic effect, we exposed the cells to the pan-caspase inhibitor zVAD prior to the nanotoxin treatment. Pre-treatment with zVAD clearly abrogated balloon-like morphology of the nanotoxin treated cells, showing a decrease in cell swelling (Fig. 3A). Consequently, cell viability was also protected by pre-treating the cell with the pan-caspase inhibitor, further indicating a caspase-dependent cell death mechanism (Fig. 3B). In agreement, zVAD pre-treatment led to a reduction of LDH release in nanotoxin treated cells (Fig. 3C). Moreover, western blotting analysis revealed that as expected, caspase-3 proteolytic activation was inhibited by zVAD pre-treatment. Hence, PARP and GSDME activation were also abolished in the zVAD pre-treated cells (Fig. 3D, Supplementary Fig. 2). Thus, pre-treatment of the cells with zVAD prior to nanotoxin exposure led to an inhibition of cell death, further corroborating the involvement of caspase-3/GSDME pathway in nanotoxin cytotoxicity. Remarkably, this is the first time that we describe the activation of this pathway by T22-PE24-H6 and T22-DITOX-H6, which opens a novel avenue for HNSCC treatment.

### Nanotoxins repeated dosage potently inhibits tumor growth in a CXCR4^+^ subcutaneous HNSCC mouse model in the absence of systemic toxicity

**Fig. 4:**
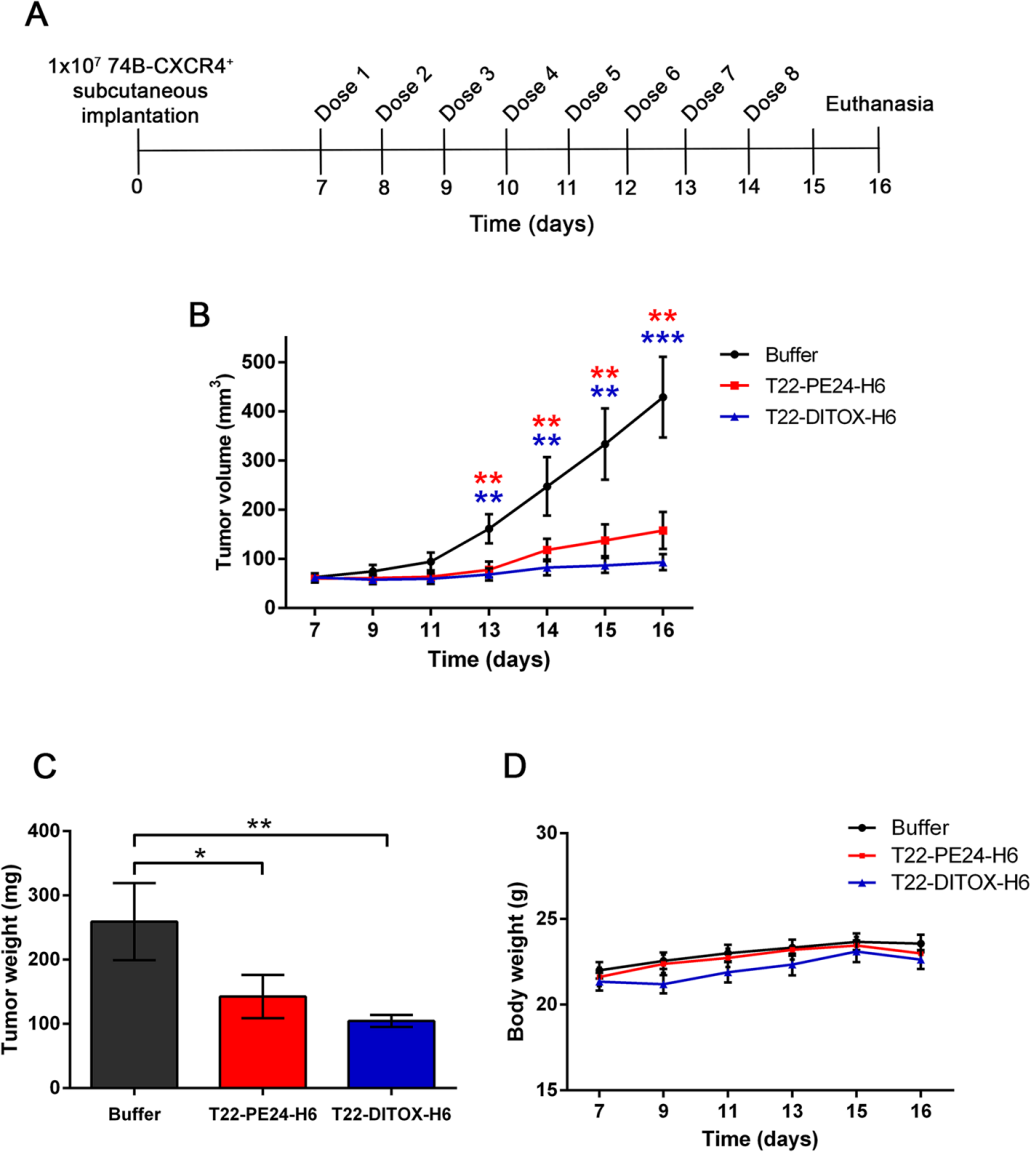
T22-PE24-H6 and T22-DITOX-H6 antitumor effect in a CXCR4^+^ HNSCC subcutaneous mouse model. A) Schematic representation of the experimental design followed in this study. **B** Variation of the tumor volume in each group (buffer, T22-PE24-H6, and T22-DITOX-H6) in the time course of the experiment. **C** Tumor weight registered at the end point of the experiment for the three experimental groups. **D** Body weight of buffer, T22-PE24-H6, and T22-DITOX-H6 treated animals along the study. * *p* < 0.05; ** *p* < 0.01; *** *p* < 0.001; *n* = 10 per group (total animal number 30). Statistical analysis performed by Student t-test. Error bars indicate SEM

The potent cytotoxic effect induced by both T22-PE24-H6 and T22-DITOX-H6 nanotoxins in the HNSCC cell lines encouraged us to evaluate their antineoplastic effect in vivo. For that, we generated a CXCR4-overexpressing subcutaneous mouse model. One week after the implantation, animals were intravenously administered buffer or 10 μg of either T22-PE24-H6 or T22-DITOX-H6 daily up to 8 doses. Tumor volume and body weight were measured on alternate days. Animals were euthanized 48 h after the last dose, and tumors were weighed and collected for later analysis, as well as different organs (Fig. 4A).

Treatment with T22-PE24-H6 and T22-DITOX-H6 nanotoxins clearly impaired tumor growth in the CXCR4-overexpressing subcutaneous tumors, as tumors from the buffer-treated animals reached bigger volumes compared to their nanotoxin-treated counterparts (Fig. 4B). Remarkably, treatment with T22-DITOX-H6 practically inhibited tumor growth, as tumor volumes did not significantly vary throughout the treatment. Consequently, tumor weight at the endpoint of the experiment was significantly higher in the tumors derived from buffer-treated mice compared to the nanotoxin-treated animals, especially the ones treated with T22-DITOX-H6 (Fig. 4C). In addition, nanotoxin treatment did not affect animal body weight in the time course of the experiment, suggesting a lack of systemic toxicity for the treatment (Fig. 4 D).

**Fig. 5:**
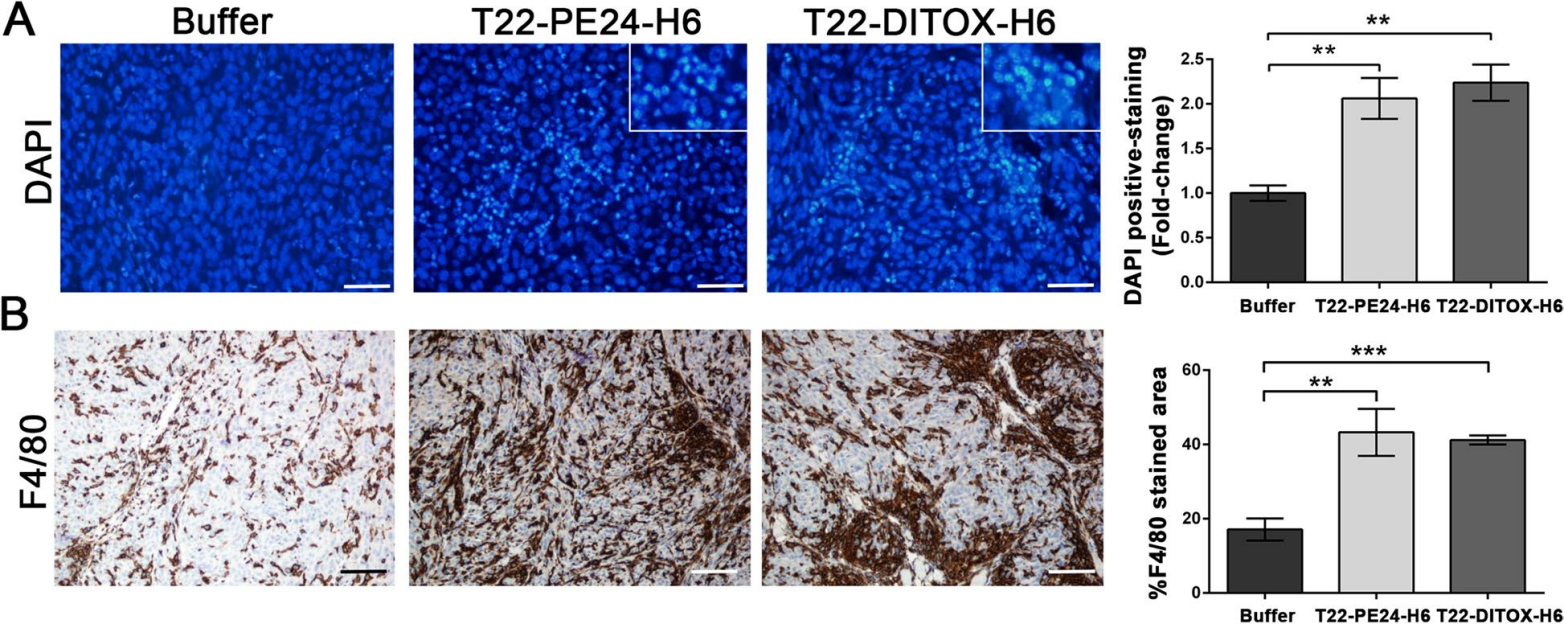
Cytotoxic effect of T22-PE24-H6 and T22-DITOX-H6 in tumor tissue. **A** Detection of dead cells by condensate DNA staining (DAPI) in the buffer, T22-PE24-H6 or T22-DITOX-H6 treated. Quantification of the number of DAPI positive stained cells in tumor tissue represented as fold-change respect to the buffer. **B** IHC analysis of tumor infiltrated macrophages in tumors from buffer, T22-PE24-H6 or T22-DITOX-H6 treated animals detected by F4/80 immunostaining. Quantification of the percentage of F4/80 positive stained cells in tumor samples from each group. Scale bars = 50 μm. F4/80 expression was quantified as mean gray value and represented as mean ± SEM. ** *p* < 0.01; *** *p* < 0.001. Statistical analysis performed by Student t-test

Tumor histology was also studied to further evaluate nanotoxin antineoplastic effect. Tumors after nanotoxin repeated treatment maintained their undifferentiated phenotype (Supplementary Fig. 3A). CXCR4 expression in tumor tissue was also maintained upon nanotoxin treatment, as no differences in percentage of positive cells were observed between groups (Supplementary Fig. 3B). Besides, nanotoxin treatment induced cell death in the tumor cells as detected by condensate DNA (DAPI staining) (Fig. 5A). The number of DAPI stained cells was found to be higher in tumor treated with either of the two nanotoxins as compared to the control group, supporting nanotoxin antitumor effect (Fig. 5A). Last but not least, we wanted to assess immune cell recruitment to the tumor site, as it is described that tumor leakage from pyroptotic cells enhances the number and activity of tumor-associated macrophages (TAMs) that phagocyte tumor cells [23]. For that, we performed a F4/80 immunohistochemistry, a well-known macrophage cell marker, finding an increase in the percentage of tumor-infiltrating macrophages in nanotoxin-treated tumors as compared to the buffer-treated ones (Fig. 5B). Altogether, these results corroborate nanotoxin potent antitumor effect, suggesting that the activation of pyroptosis in tumor cells also enhances immune cell recruitment to the tumor site, further contributing to the antitumor effect.

**Fig. 6:**
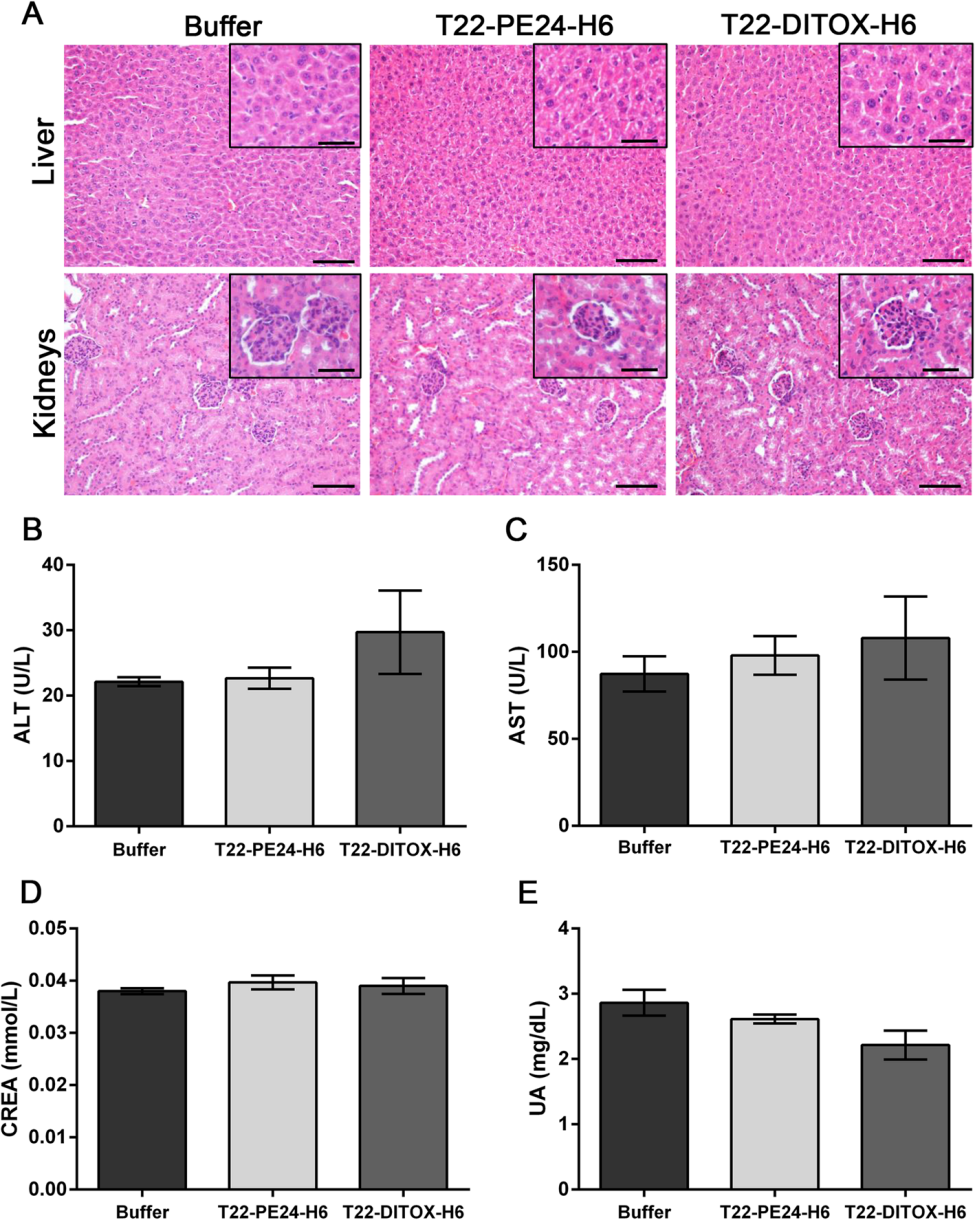
T22-PE24-H6 and T22-DITOX-H6 toxicity assessment. **A** Histopathological analysis by H&E staining in liver and kidneys samples from buffer, T22-PE24-H6 or T22-DITOX-H6 treated animals. **B**, **C**, **D**, and **E** Oxaloacetic transaminase (GOT) (**B**), and glutamic pyruvic transaminase (GPT) (C) enzyme activities, as well as creatinine (**D**) and uric acid (**E**) levels in plasma samples from buffer, T22-PE24-H6 or T22-DITOX-H6 treated animals. Scale bars = 100 μm and 50 μm (zoom in). Statistical analysis performed by Student t-test. Error bars indicate SEM

Importantly, no off-target toxicity was observed upon nanotoxin treatment, neither in the T22-PE24-H6 nor in the T22-DITOX-H6 group. We assessed nanotoxin toxicity by histopathology (H&E staining) in liver and kidneys tissue, organs involved in the metabolism and elimination of drugs. As it could be observed by two independent observers, no histological alterations were detected in either of the two organs, which presented their normal architecture and morphology (Fig. 6A). Moreover, to further corroborate treatment’s lack of toxicity, hepatic and renal function were studied by hepatic transaminases activity, as well as creatinine and uric acid levels in plasma. Results showed no statistically significant differences in transaminases activity, nor in creatinine or uric acid concentrations in plasma between control and nanotoxin treated animals (Fig. 6B-E). In addition, these values were between the normal range of a healthy animal [29]. Thus, T22-PE24-H6 and T22-DITOX-H6 nanotoxin treatment at the chosen administration conditions, does not induce off-target toxicity in the CXCR4^+^ HNSCC subcutaneous mouse model.

Moreover, to evaluate the long-term toxicity induced by nanotoxin treatment, animals received the same dosage as in the antitumor effect experiment, but were further maintained for 4 weeks after the end of the treatment (Supplementary Fig. 4A). Importantly, non-tumor bearing mice were utilized, as the lack of tumor and consequently the absence of nanotoxin uptake by tumor tissue, will potentially increase nanotoxin concentration in the bloodstream, thus providing a better evaluation of their potential off-target toxicity. Remarkably, none of the nanotoxins, neither T22-PE24-H6 nor T22-DITOX-H6, shortly after their administration at repeated doses or 4 weeks after the end of the treatment, induced any changes in animal body weight (Supplementary Fig. 4B). In addition, cell blood count (CBC) analyses were performed weekly to assess treatment derived toxicity. No differences between buffer and nanotoxin-treated animals were observed in terms of white blood cells (white blood cell count (WBC), neutrophils, lymphocytes, monocytes, eosinophils, and basophils), red blood cells (red blood cell count (RCB), hemoglobin (HGB), hematocrit (HCT), mean cell volume (MCV), mean corpuscular hemoglobin (MCH), mean corpuscular hemoglobin concentration (MCHC), and red blood cell distribution width (RDW)), or platelets (platelet count, mean platelet volume (MPV), platelet distribution width (PDW), and plateletcrit (PCT)) (Supplementary Fig. 5). Moreover, these values were between the normal range for a healthy Swiss nude mouse. Lastly, no histopathological alterations were observed in liver, kidneys, spleen, or bone marrow, implying that nanotoxin treatment did not induce any long-term toxic effects in the animals (Supplementary Fig. 4C). Thus, the lack of change in mouse body weight, together with unaltered CBCs, and the absence of histological alterations in non-tumor tissues, indicate a lack of long-term toxicity by the nanotoxins when administered at a dosage that achieves a highly significant antitumor effect.

### GSDME and CXCR4 expression in HNSCC and clinical implications

**Fig. 7:**
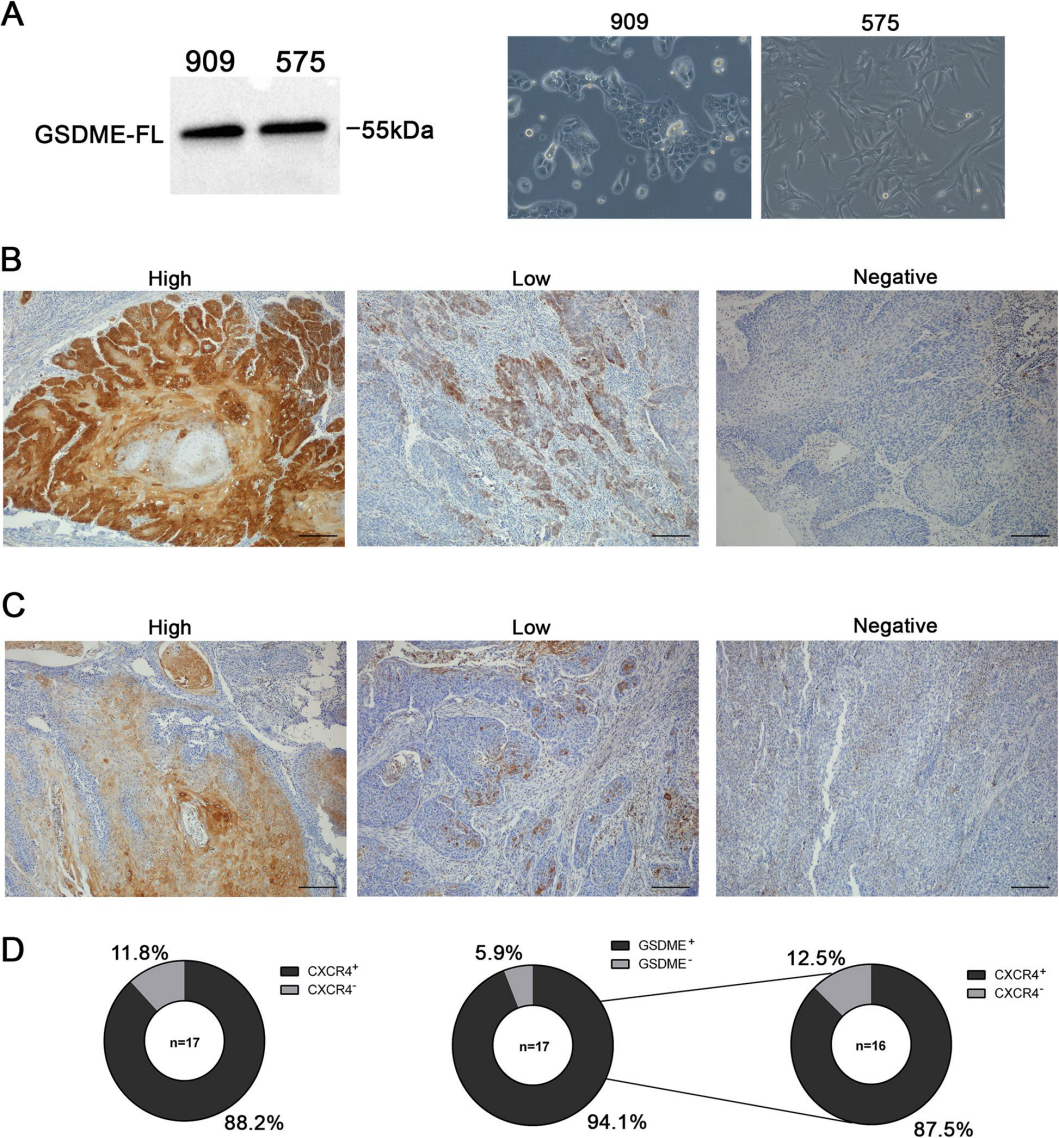
GSDME expression levels in HNSCC patient samples. **A** GSDME expression analysis by western blotting in two patient derived tumor samples (909 and 575) maintained in vitro. Phase-contrast imaging of 909 and 575 cultures showing their morphology (magnification 400x). **B** Representative IHC images of GSDME expression in HNSCC patient tumor samples presenting different levels of expression. **C** Representative IHC images of CXCR4 receptor expression in patient tumor samples. **D** Percentage of CXCR4 and GSDME positive and negative stained samples in the HNSCC patient cohort. Percentage of GSDME^+^ samples that were also positive for CXCR4 IHC. Scale bars = 200 μm

Taking into consideration these findings, we wanted to evaluate the clinical relevance of GSDME activation in HNSCC patients. Given that GSDME presents a tumor suppressive role, it has been reported that GSDME inactivation is a strategy developed by cancer cells to avoid cell death. However, both HPV^−^ HNSCC cells lines used in this study expressed GSDME, and this protein was able to exert its activity upon nanotoxin treatment. Moreover, two patient-derived samples obtained from CXCR4^+^ tumors maintained in vitro also expressed the protein (Fig. 7A). Thus, to further investigate GSDME expression in HNSCC, we conducted an analysis using data from The Cancer Genome Atlas (TCGA) with the UALCAN analysis software [30]. First, we found that CXCR4 is overexpressed in tumoral tissue compared to healthy tissue (Supplementary Fig. 6). Similarly, DFNA5 (gene encoding GSDME) was also overexpressed in tumors compared to non-tumor tissue (Supplementary Fig. 7A). Moreover, DFNA5 was more expressed in HPV^−^ tumors compared to the HPV^+^ ones (Supplementary Fig. 7B). Interestingly, high DFNA5 expression in tumor tissue correlated with worse overall survival (OS) (Supplementary Fig. 7C). These results prompted us to perform a IHC analysis of GSMDE expression in a cohort of 17 HNSCC patients (Fig. 7B). First, we assessed the CXCR4 expression in the HNSCC samples finding that 88.2% were positive for the receptor (Fig. 7C). Moreover, 94.1% of these patient samples presented positive GSDME staining, independently of their prognosis (Fig. 7C). Importantly, 87.5% of the patient samples expressing GSDME were also positive for CXCR4 immunostaining (Fig. 7C). This subset of patients could potentially benefit from a CXCR4-targeted treatment capable of activating GSDME, such as the nanotoxins T22-PE24-H6 and T22-DITOX-H6. Thus, we found that GSDME is widely expressed in HNSCC patients highlighting the relevance of GSDME-dependent pyroptosis in disease course and opening a novel avenue for future treatments.

## Discussion

In this study (which rationale is summarized in Supplementary Fig. 8), we report for the first time that targeted toxin delivery to CXCR4-overexpresing (CXCR4^+^) human HNSCC cells, achieved by the T22-PE24-H6 and T22-DITOX-H6 nanotoxins, activates caspase-3/GSDME-dependent pyroptosis in CXCR4^+^ cancer cells. Consequently, nanotoxin treatment leads to a potent blockade of tumor growth in a HNSCC model, without inducing systemic toxicity. Importantly, activation of this cell death mechanism alternative to apoptosis is expected to overcome the low response rate to chemotherapy and radiotherapy in HNSCC patients [5, 31].

We meticulously demonstrate that both nanotoxins activate caspase-3/GSDME-dependent pyroptosis in CXCR4^+^ human HNSCC cell lines. First, we have reported that, upon nanotoxin treatment, HNSCC cell lines displayed distinctive pyroptotic features, including balloon-like morphology, increase in LDH release, and lytic cell death. These observations associated with caspase-3 activation followed by GSDME cleavage to generate the pyroptotic effector pore inductor GSDME N-terminus, as detected by immunoblotting. Importantly, pre-treatment with the pan-caspase inhibitor zVAD blocked all the above described pyroptotic features. Thus, the selective bacterial exotoxin release in CXCR4^+^ HNSCC cells activate caspase-3/GSDME switch from apoptosis to pyroptosis, as described for different untargeted and targeted drugs in other cancer types [24, 25, 32–34].

In addition, and according to the potent cytotoxicity (low nanomolar IC_50_) observed in vitro, repeated intravenous administration of each nanotoxin in a subcutaneous CXCR4^+^ HNSCC mouse model, induced a potent blockade of tumor growth, flattening the growth curves, especially for the T22-DITOX-H6 nanotoxin, in the absence of systemic toxicity or adverse effects. This high therapeutic window is most likely due to the strict CXCR4-dependent killing achieved by the nanostructured toxins. T22-PE24-H6 and T22-DITOX-H6 internalize exclusively in CXCR4^+^ cells, releasing their toxin domains by furin cleavage upon cell internalization, leading to EF-2 inactivation and protein synthesis inhibition that results in cell death [19]. This effect was demonstrated in vitro in two different HNSCC cell lines, showing that only CXCR4^+^ cells are killed by the nanotoxins and also by the demonstration of a complete cell death blockade by AMD3100 (CXCR4 antagonist) exposure prior to nanotoxins treatment.

Consistently, our previous work supports the selective biodistribution of the nanotoxins to the high CXCR4-overexpressing tumor tissue, avoiding the accumulation and off-target toxicity in normal organs with low or negligible CXCR4 expression. In this context, we have recently reported this high tumor selectivity using a CXCR4-targeted fluorescent nanocarrier, that displays the exact self-assembling nanoparticle structure of the nanotoxins, in HNSCC, colorectal cancer [35], and lymphoma [36] models. Their size above the 7 nm renal filtration cut-off allows a high recirculation in the bloodstream, while the multivalency derived from the display of multiple T22 peptide domains enables superselectivity [18] regarding CXCR4^+^ target cell internalization, which exploits the CXCR4 overexpression in tumor compared to normal tissue.

Moreover, innate antitumor immunity may have contributed to the anticancer activity based on the dramatic increase in tumor-infiltrating macrophages (TAM) found in nanotoxin-treated animals. It has been reported that GSDME activation enhances antitumor effect by stimulating TAM phagocytosis, as well as NKs and CD8^+^ T lymphocytes activation [6, 23, 37]. Although preliminary, these findings may suggest an activation of the pyroptotic pathway in vivo upon treatment, leading to an enhanced immune cell recruitment to the tumor site.

Importantly, different studies have described that conventional chemotherapy threatening off-target toxicities are induced by the activation of GSDME mediated pyroptosis in non-tumor cells, as these agents lack tumor targeting capacity [24, 38, 39]. In contrast, our CXCR4-targeted nanotoxins, administered at a repeated dosage schedule, did not induce acute systemic toxicity (at the end of treatment) nor long-term toxicity (4 weeks after treatment), in the HNSCC mouse model, displaying undetectable markers of toxicity in plasma, unaltered cell blood count and lack of histological alterations in non-tumor organs, at a dosage that achieves a potent antitumor effect. Thus, a strict targeted drug delivery to cancer cells is crucial to prevent GSDME activation in healthy tissues.

Regarding the clinical relevance of our results, it is important to highlight that apoptosis avoidance is the main mechanism of resistance to radiotherapy, cisplatin, 5-flourouracil or docetaxel/paclitaxel, leading to treatment failure and patient death [3, 5, 6, 20]. Moreover, CXCR4 overexpression, in HNSCC and other neoplasias, also associates with resistance, relapse and metastatic potential [7, 8, 40]. Thus, our nanotoxin approach aims to overcome tumor resistance and increase cure rates in HNSCC, by switching cell death induction from apoptosis to caspase-3/GSDME-dependent pyroptosis, as pursued in other cancer types [34, 37, 41].

Interestingly, a large percent of HNSCC patients could be candidates to targeted nanotoxin therapy since in our 17-patient cohort, 88.2% were positive for the CXCR4 receptor, 94% of them expressed GSDME in tumor tissue, whereas 87% of GSDME^+^ tumors also co-expressed CXCR4. Moreover, we have also studied CXCR4 expression in two different cell cultures derived from HNSCC patient tumor samples, named as 575 and 909. Interestingly, although both patient samples presented a strong CXCR4 expression, when cultured in vitro, 575 and 909 cell cultures lost their CXCR4 expression with successive culture passages. Remarkably, when re-inoculated in vivo in immunodeficient mice, the generated tumors expressed again CXCR4 (Supplementary Fig. 9). These results demonstrate that CXCR4 expression is downregulated in vitro, suggesting that CXCR4 regulation plays an important role in tumor progression in vivo. In addition, our TGCA analysis of HNSCC samples showed that GSDME overexpression in tumor compared to normal tissue, correlates with worse OS, which may be explained by the reported lack of tumor infiltrated lymphocytes in GSDME^+^ HNSCC tumors by other authors [42]. Consistently with our results, GSDME overexpression in tumor tissue has also been reported in different cancer types [25, 32, 43, 44], which contrasts with the assumption that GSDME is silenced in tumors given its tumor suppressor role [23, 24, 45–49]. In any case, it is clear that CXCR4 and GSDME markers could be used to select HNSCC patients that might benefit from our nanotoxin therapy.

So far, most of the targeted drugs to treat resistant HNSCC have failed at early stages. Immunotoxins based on *Pseudomonas* exotoxin that target mesothelin in HNSCC stopped development in early clinical trials [50]. Similarly, ADCs targeting different surface markers (EGFR, c-MET, HER2, etc.) conjugated to the PBD toxin or the microtubule inhibitor Auristatin, did not progress due to toxicity concerns [14]. Only the EGFR inhibitor cetuximab and the PD-1/PD-L1 immune checkpoint inhibitors pembrolizumab, nivolumab, and durvalumab, improve survival in recurrent or metastatic HNSCC [51]. We believe that T22-PE24-H6 and T22-DITOX-H6 nanotoxins present important features that will exceed the performance of current immunotoxins and ADCs. These nanotoxins are formed by self-assembly of multiple monomers, displaying numerous targeting ligands that confer superselectivity, and the ability to incorporate multiple cytotoxic domains into a single nanotoxin (Supplementary Fig. 8). These facts contrast with immunotoxins or ADCs, that display only one ligand per molecule, a lower cytotoxic payload, and show drug leakage during circulation inducing off-target effects [12, 13, 15–17].

## Conclusions

In summary, the activation of caspase-3/GSDME-dependent pyroptosis by nanostructured toxins targeting CXCR4 opens a novel and virtually unexplored therapeutic approach for HNSCC or other cancer types. Thus, T22-PE24-H6 and T22-DITOX-H6 nanotoxin treatments may turn sensitive the recurrent or metastatic HNSCC because of their ability to trigger a cell death mechanism alternative to apoptosis, since apoptosis blockade is the main mechanism of resistance to currently used chemotherapy and radiotherapy in HNSCC patients [5, 6]. Additionally, the nanotoxin switch from non-inflammatory apoptosis to the inflammatory pyroptosis may also engage immune cells that could enhance anti-tumor immunity.

## Supplementary Information

**Additional file 1.**

## Data Availability

The datasets used and/or analysed during the current study are available from the corresponding author on reasonable request.

## Abbreviations

HNSCC: Head and neck squamous cell carcinoma
CXCR4: Chemokine receptor 4
GSDME: Gasdermin E
LDH: Lactate dehydrogenase
ADC: Antibody-drug conjugate
IL-2: Interleukin-2
PBD: Pyrrolobenzodiazepine
HPV: Human papilloma virus
DMEM: Dulbecco’s Modified Eagle’s Medium
FBS: Fetal Bovine Serum
PI: Propidium iodide
WB: Western Blotting
SPF: Specific pathogen-free
IHC: Immunohistochemistry
GOT: Glutamic oxaloacetic transaminase
GPT: Glutamic pyruvic transaminase
SD: Standard deviation
SEM: Standard error
TAM: Tumor-associated macrophage
H&E: Hematoxylin-eosin
WBC: White blood cell count
RCB: Red blood cell count
HGB: Hemoglobin
HCT: Hematocrit
MCV: Mean cell volume
MCH: Mean corpuscular hemoglobin
MCHC: Mean corpuscular hemoglobin concentration
RDW: Red blood cell distribution width
MPV: Mean platelet volume
PDW: Platelet distribution width
PCT: Plateletcrit

## References

[1] Sacco AG, Cohen EE (2015). Current treatment options for recurrent or metastatic head and neck squamous cell carcinoma. J Clin Oncol.

[2] Johnson DE, Burtness B, Leemans CR, Lui VWY, Bauman JE, Grandis JR (2020). Head and neck squamous cell carcinoma. Nat Rev Dis Prim.

[3] Picon H, Guddati AK (2020). Mechanisms of resistance in head and neck cancer. Am J Cancer Res.

[4] López-Verdín S, Lavalle-Carrasco J, Carreón-Burciaga RG, Serafín-Higuera N, Molina-Frechero N, González-González R (2018). Molecular markers of anticancer drug resistance in head and neck squamous cell carcinoma: a literature review. Cancers (Basel)..

[5] Kanno Y, Chen C-Y, Lee H-L, Chiou J-F, Chen Y-J (2021). Molecular mechanisms of chemotherapy resistance in head and neck cancers. Front Oncol.

[6] Raudenská M, Balvan J, Masařík M (2021). Cell death in head and neck cancer pathogenesis and treatment. Cell Death Dis.

[7] León X, Diez S, García J, Lop J, Sumarroca A, Quer M (2016). Expression of the CXCL12/CXCR4 chemokine axis predicts regional control in head and neck squamous cell carcinoma. Eur Arch Otorhinolaryngol.

[8] De-Colle C, Menegakis A, Mönnich D, Welz S, Boeke S, Sipos B (2018). SDF-1/CXCR4 expression is an independent negative prognostic biomarker in patients with head and neck cancer after primary radiochemotherapy. Radiother Oncol.

[9] Pastan I, Hassan R, Fitzgerald DJ, Kreitman RJ (2006). Immunotoxin therapy of cancer. Nat Rev Cancer England..

[10] Alewine C, Hassan R, Pastan I (2015). Advances in anticancer immunotoxin therapy. Oncologist.

[11] Wu T, Zhu J (2021). Recent development and optimization of *pseudomonas aeruginosa* exotoxin immunotoxins in cancer therapeutic applications. Int Immunopharmacol.

[12] Vallera DA, Kreitman RJ (2018). Immunotoxins targeting B cell malignancy-Progress and problems with immunogenicity. Biomedicines..

[13] Kim J-S, Jun S-Y, Kim Y-S (2020). Critical issues in the development of immunotoxins for anticancer therapy. J Pharm Sci.

[14] Perrotti V, Caponio VCA, Mascitti M, Lo Muzio L, Piattelli A, Rubini C (2021). Therapeutic potential of antibody-drug conjugate-based therapy in head and neck cancer: a systematic review. Cancers (Basel)..

[15] Beck A, Goetsch L, Dumontet C, Corvaïa N (2017). Strategies and challenges for the next generation of antibody-drug conjugates. Nat Rev Drug Discov.

[16] Wolska-Washer A, Robak T (2019). Safety and tolerability of antibody-drug conjugates in Cancer. Drug Saf.

[17] Masters JC, Nickens DJ, Xuan D, Shazer RL, Amantea M (2018). Clinical toxicity of antibody drug conjugates: a meta-analysis of payloads. Invest New Drugs.

[18] Liu M, Apriceno A, Sipin M, Scarpa E, Rodriguez-Arco L, Poma A (2020). Combinatorial entropy behaviour leads to range selective binding in ligand-receptor interactions. Nat Commun.

[19] Sánchez-García L, Serna N, Álamo P, Sala R, Céspedes MV, Roldan M (2018). Self-assembling toxin-based nanoparticles as self-delivered antitumoral drugs. J Control Release.

[20] Holohan C, Van Schaeybroeck S, Longley DB, Johnston PG (2013). Cancer drug resistance: an evolving paradigm. Nat Rev Cancer.

[21] Brenner JC, Graham MP, Kumar B, Saunders LM, Kupfer R, Lyons RH (2010). Genotyping of 73 UM-SCC head and neck squamous cell carcinoma cell lines. Head Neck.

[22] Rioja-Blanco E, Arroyo-Solera I, Álamo P, Casanova I, Gallardo A, Unzueta U, et al. Self-assembling protein nanocarrier for selective delivery of cytotoxic polypeptides to CXCR4+ head and neck squamous cell carcinoma tumors. Acta Pharm Sin B. 2021; Available from: https://www.sciencedirect.com/science/article/pii/S2211383521003919. 10.1016/j.apsb.2021.09.030 PMC9136533 35646535

[23] Zhang Z, Zhang Y, Xia S, Kong Q, Li S, Liu X (2020). Gasdermin E suppresses tumour growth by activating anti-tumour immunity. Nature.

[24] Wang Y, Gao W, Shi X, Ding J, Liu W, He H (2017). Chemotherapy drugs induce pyroptosis through caspase-3 cleavage of a gasdermin. Nature.

[25] Lu H, Zhang S, Wu J, Chen M, Cai MC, Fu Y (2018). Molecular targeted therapies elicit concurrent apoptotic and GSDME-dependent pyroptotic tumor cell death. Clin Cancer Res.

[26] Zhang X, Zhang P, An L, Sun N, Peng L, Tang W (2020). Miltirone induces cell death in hepatocellular carcinoma cell through GSDME-dependent pyroptosis. Acta Pharm Sin B.

[27] Zhang Y, Yang J, Wen Z, Chen X, Yu J, Yuan D (2020). A novel 3′,5′-diprenylated chalcone induces concurrent apoptosis and GSDME-dependent pyroptosis through activating PKCδ/JNK signal in prostate cancer. Aging (Albany NY).

[28] Kong Y, Feng Z, Chen A, Qi Q, Han M, Wang S (2019). The natural flavonoid Galangin elicits apoptosis, Pyroptosis, and autophagy in glioblastoma. Front Oncol.

[29] Cañes L, Martí-Pàmies I, Ballester-Servera C, Alonso J, Serrano E, Briones AM (2021). High NOR-1 (neuron-derived orphan receptor 1) expression strengthens the Vascular Wall response to angiotensin II leading to aneurysm formation in mice. Hypertens (Dallas, Tex 1979).

[30] Chandrashekar DS, Bashel B, Balasubramanya SAH, Creighton CJ, Ponce-Rodriguez I, Chakravarthi BVSK (2017). UALCAN: a portal for facilitating tumor subgroup gene expression and survival analyses. Neoplasia.

[31] Wang L, Qin X, Liang J, Ge P (2021). Induction of Pyroptosis: a promising strategy for Cancer treatment. Front Oncol.

[32] Zhang C-C, Li C-G, Wang Y-F, Xu L-H, He X-H, Zeng Q-Z (2019). Chemotherapeutic paclitaxel and cisplatin differentially induce pyroptosis in A549 lung cancer cells via caspase-3/GSDME activation. Apoptosis.

[33] Tsuchiya K (2021). Switching from apoptosis to Pyroptosis: Gasdermin-elicited inflammation and antitumor immunity. Int J Mol Sci..

[34] Jiang M, Qi L, Li L, Li Y (2020). The caspase-3/GSDME signal pathway as a switch between apoptosis and pyroptosis in cancer. Cell Death Discov.

[35] Céspedes MV, Unzueta U, Tatkiewicz W, Sánchez-Chardi A, Conchillo-Solé O, Álamo P (2014). In vivo architectonic stability of fully de novo designed protein-only nanoparticles. ACS Nano.

[36] Falgàs A, Pallarès V, Unzueta U, Céspedes MV, Arroyo-Solera I, Moreno MJ (2020). A CXCR4-targeted nanocarrier achieves highly selective tumor uptake in diffuse large B-cell lymphoma mouse models. Haematologica.

[37] Yu P, Zhang X, Liu N, Tang L, Peng C, Chen X (2021). Pyroptosis: mechanisms and diseases. Signal Transduct Target Ther.

[38] Wang Y-Y, Liu X-L, Zhao R (2019). Induction of Pyroptosis and its implications in Cancer management. Front Oncol.

[39] Huang Z, Zhang Q, Wang Y, Chen R, Wang Y, Huang Z (2020). Inhibition of caspase-3-mediated GSDME-derived pyroptosis aids in noncancerous tissue protection of squamous cell carcinoma patients during cisplatin-based chemotherapy. Am J Cancer Res.

[40] Chatterjee S, Behnam Azad B, Nimmagadda S (2014). The intricate role of CXCR4 in cancer. Adv Cancer Res.

[41] Wu D, Wang S, Yu G, Chen X (2021). Cell death mediated by the Pyroptosis pathway with the aid of nanotechnology: prospects for Cancer therapy. Angew Chem Int Ed Engl.

[42] Liu Z, Liu H, Dong Q, Li H, Zhang B, Liu Y (2020). Prognostic role of DFNA5 in head and neck squamous cell carcinoma revealed by systematic expression analysis.

[43] Wu M, Wang Y, Yang D, Gong Y, Rao F, Liu R (2019). A PLK1 kinase inhibitor enhances the chemosensitivity of cisplatin by inducing pyroptosis in oesophageal squamous cell carcinoma. EBioMedicine.

[44] Zhou B, Zhang J-Y, Liu X-S, Chen H-Z, Ai Y-L, Cheng K (2018). Tom20 senses iron-activated ROS signaling to promote melanoma cell pyroptosis. Cell Res.

[45] Xia X, Wang X, Cheng Z, Qin W, Lei L, Jiang J, et al. The role of pyroptosis in cancer: pro-cancer or pro-“host”? Cell Death Dis. 2019;10. 10.1038/s41419-019-1883-8. 10.1038/s41419-019-1883-8 PMC6733901 31501419

[46] Lage H, Helmbach H, Grottke C, Dietel M, Schadendorf D (2001). DFNA5 (ICERE-1) contributes to acquired etoposide resistance in melanoma cells. FEBS Lett.

[47] Kim MS, Chang X, Yamashita K, Nagpal JK, Baek JH, Wu G (2008). Aberrant promoter methylation and tumor suppressive activity of the DFNA5 gene in colorectal carcinoma. Oncogene.

[48] Akino K, Toyota M, Suzuki H, Imai T, Maruyama R, Kusano M (2007). Identification of DFNA5 as a target of epigenetic inactivation in gastric cancer. Cancer Sci.

[49] Kim MS, Lebron C, Nagpal JK, Chae YK, Chang X, Huang Y (2008). Methylation of the DFNA5 increases risk of lymph node metastasis in human breast cancer. Biochem Biophys Res Commun.

[50] Hassan R, Ho M (2008). Mesothelin targeted cancer immunotherapy. Eur J Cancer.

[51] Cramer JD, Burtness B, Le QT, Ferris RL (2019). The changing therapeutic landscape of head and neck cancer. Nat Rev Clin Oncol.

