# Supplementary material for: CXCR4-targeted nanotoxins induce GSDME-dependent pyroptosis in head and neck squamous cell carcinoma
Source: J Exp Clin Cancer Res. 2022 Feb 4;41:49. doi: 10.1186/s13046-022-02267-8 (PMC8815235; doi:10.1186/s13046-022-02267-8)
Supplement: Supplementary file 1 — Additional file 1. [file 13046_2022_2267_MOESM1_ESM.docx]

**Supplementary data**

**
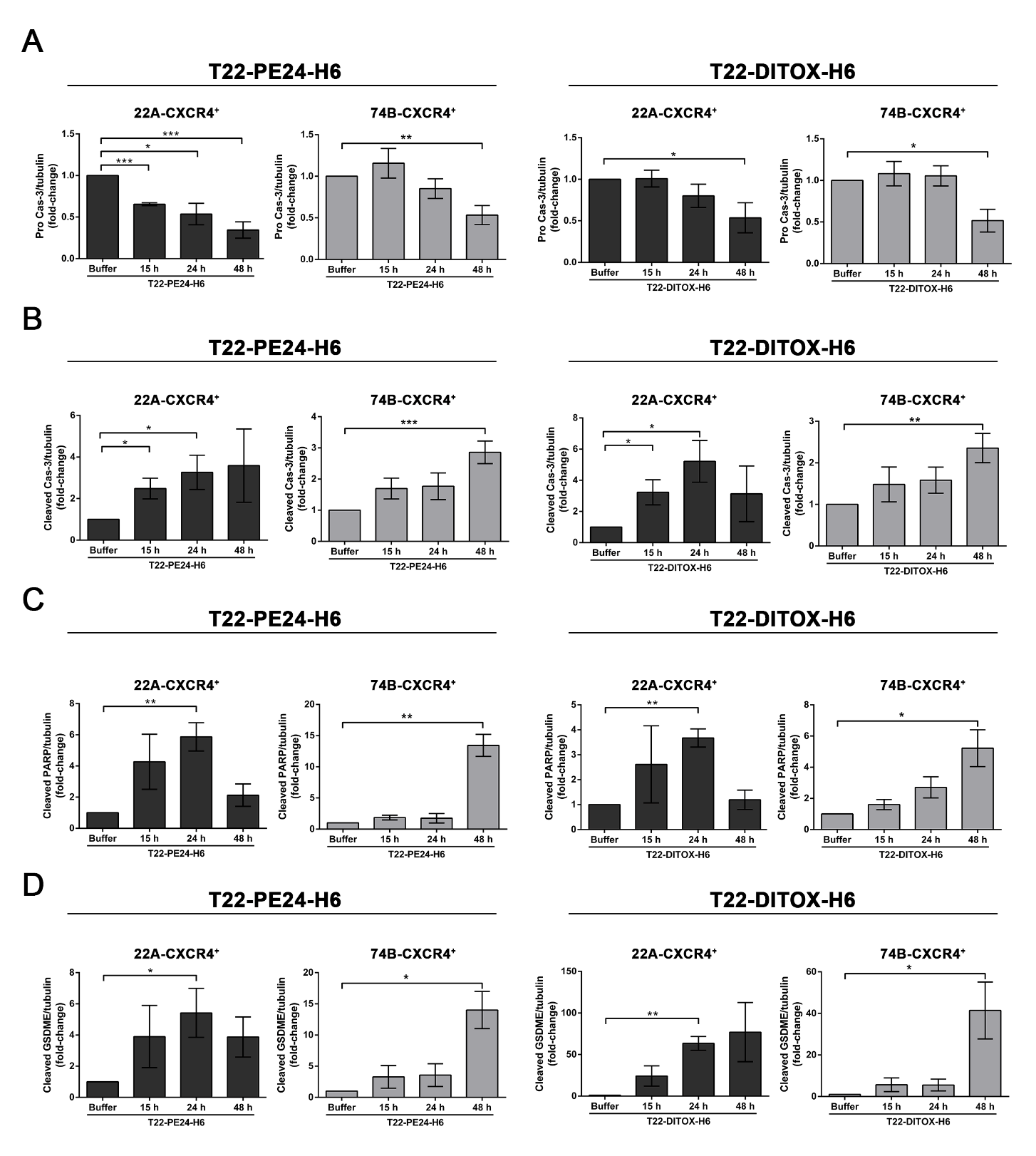
**

**Supplementary figure 1. Quantitation of relative protein intensity for different pyroptotic markers after nanotoxin treatment.** Pro-caspase-3 (A), cleaved caspase-3 (B), cleaved PARP (C), and cleaved GSDME (D) western blotting intensity quantification from protein extracts from 22A-CXCR4^+^ and 74B-CXCR4^+^ cell lines treated with T22-PE24-H6 and T22-DITOX-H6 for 15 h, 24 h, and 48 h. * p<0.05; ** p<0.01; *** p<0.001. Each column represents the mean value of at least three biological replicates. Statistical analysis performed by Student t-test. Error bars indicate SEM.

**
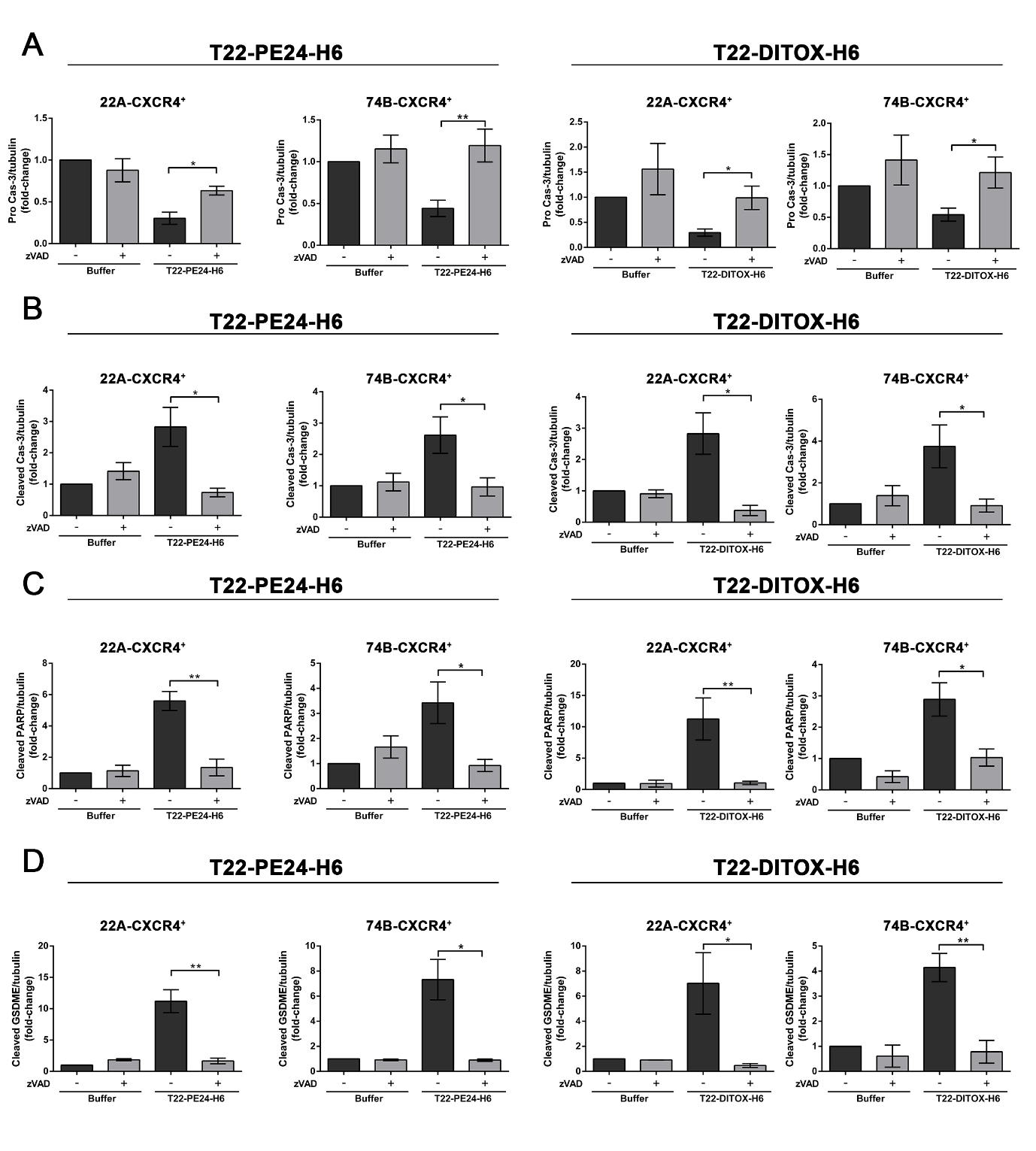
**

**Supplementary figure 2. Quantitation of relative protein intensity of different pyroptotic markers in HNSCC cell lines pre-treated with the pan-caspase inhibitor zVAD before nanotoxin treatment.** Pro-caspase-3 (A), cleaved caspase-3 (B), cleaved PARP (C), and cleaved GSDME (D) western blotting intensity quantification from protein extracts from 22A-CXCR4^+^ and 74B-CXCR4^+^ cell lines pre-treated with zVAD prior to the nanotoxin treatment for 15 h, 24 h, and 48 h. * p<0.05; ** p<0.01. Each column represents the mean value of at least three biological replicates. Statistical analysis performed by Student t-test. Error bars indicate SEM.

**
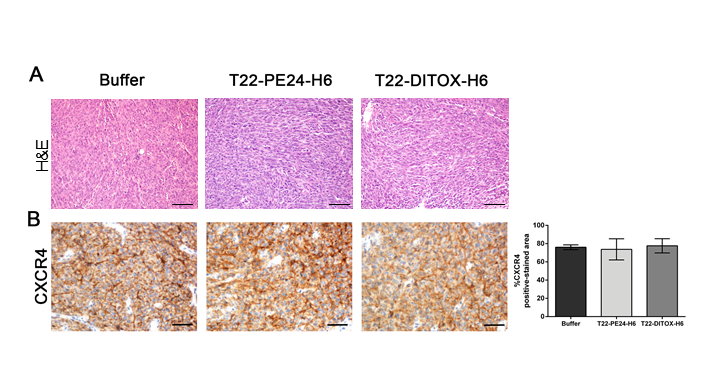
**

**Supplementary figure 3. Histological analysis of the subcutaneous tumors obtained at the end of the repeated dose nanotoxin treatment.** A) H&E images from the buffer, T22-PE24-H6, and T22-DITOX-H6 tumors. B) Representative IHC images of the CXCR4 expression in tumor samples from buffer, T22-PE24-H6, and T22-DITOX-H6 treated animals. Quantification of the percentage of CXCR4 positive stained cells in tumors from each group. Scale bars = 100µm and 50µm. CXCR4 expression was quantified as mean gray value and represented as mean ± SEM.


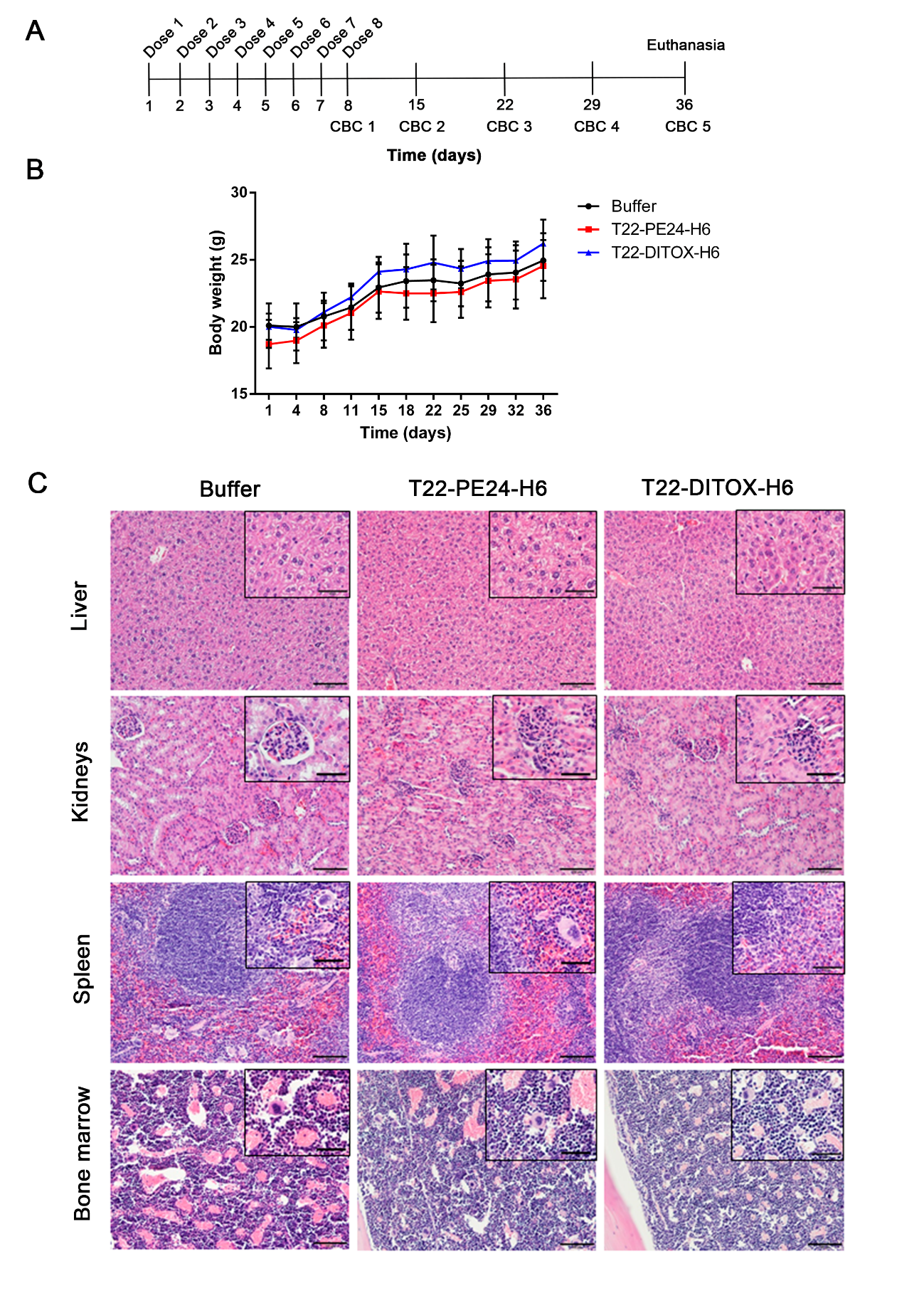


**Supplementary figure 4. T22-PE24-H6 and T22-DITOX-H6 lack long-term off-target toxicity in non-tumor tissues.** A) Schematic representation of the experimental procedure conducted in this study. B) Variation of the body weight in each group (buffer, T22-PE24-H6, and T22-DITOX-H6) along the time course of the experiment. C) Representative H&E images of liver, kidneys, spleen, and bone marrow from buffer, T22-PE24-H6, and T22-DITOX-H6 treated animals. Scale bars = 100µm and 50µm (zoom in). Statistical analysis performed by Student t-test. Error bars indicate SEM.


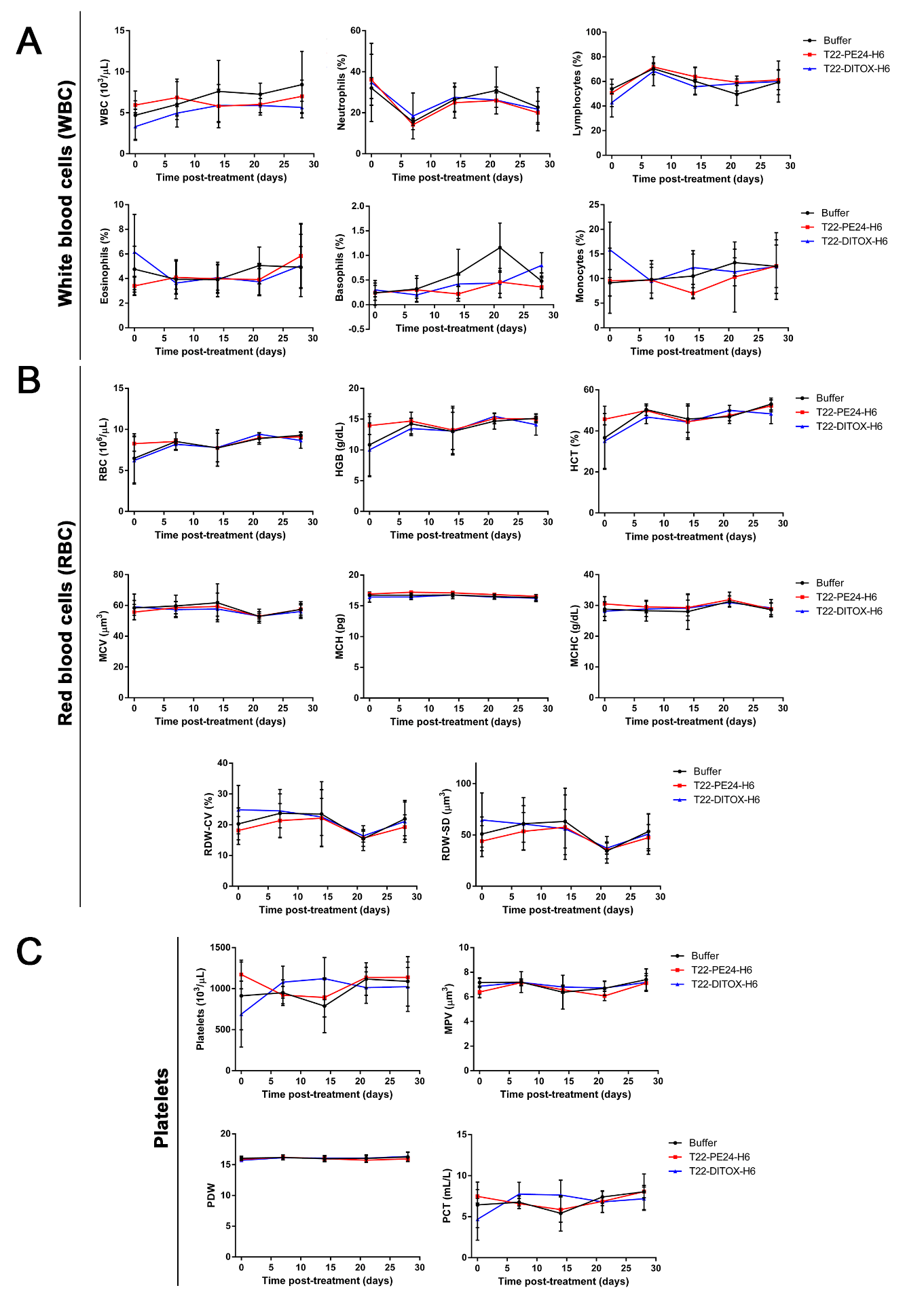


**Supplementary figure 5. Cell blood count (CBC) analysis to evaluate long-term toxicity after repeated T22-PE24-H6 or T22-DITOX-H6 administration.** A) Evaluation of total white blood cells (WBC), neutrophils, lymphocytes, eosinophils, basophils, and monocytes in each experimental group (buffer, T22-PE24-H6, and T22-DITOX-H6) through the time course of the experiment. B) Red blood cells (RBC) and RBC indices analyses (hemoglobin (HGB), hematocrit (HCT), mean cell volume (MCV), mean corpuscular hemoglobin (MCH), mean corpuscular hemoglobin concentration (MCHC), and red blood cells distribution width (RDW)) from buffer and nanotoxin treatment mice during the experiment. C) Platelets and platelet indices (mean platelet volume (MPV), platelet distribution width (PDW), and plateletcrit (PCT)) registered throughout the experiment in each experimental group. n=5 per group (total animal number 15). Statistical analysis performed by Student t-test. Error bars indicate SD.


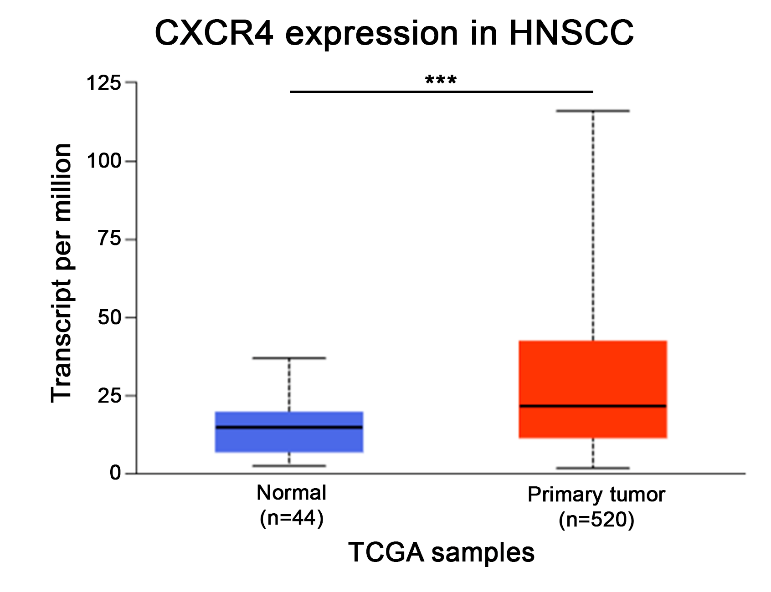


**Supplementary figure 6. TCGA analysis of the expression of CXCR4 in HNSCC patients.** Transcriptomic analysis of the expression of CXCR4 in normal tissue compared to primary tumor samples from HNSCC patients. Analysis was performed using data from the TCGA with the UALCAN analysis software. *** p<0.001.

**
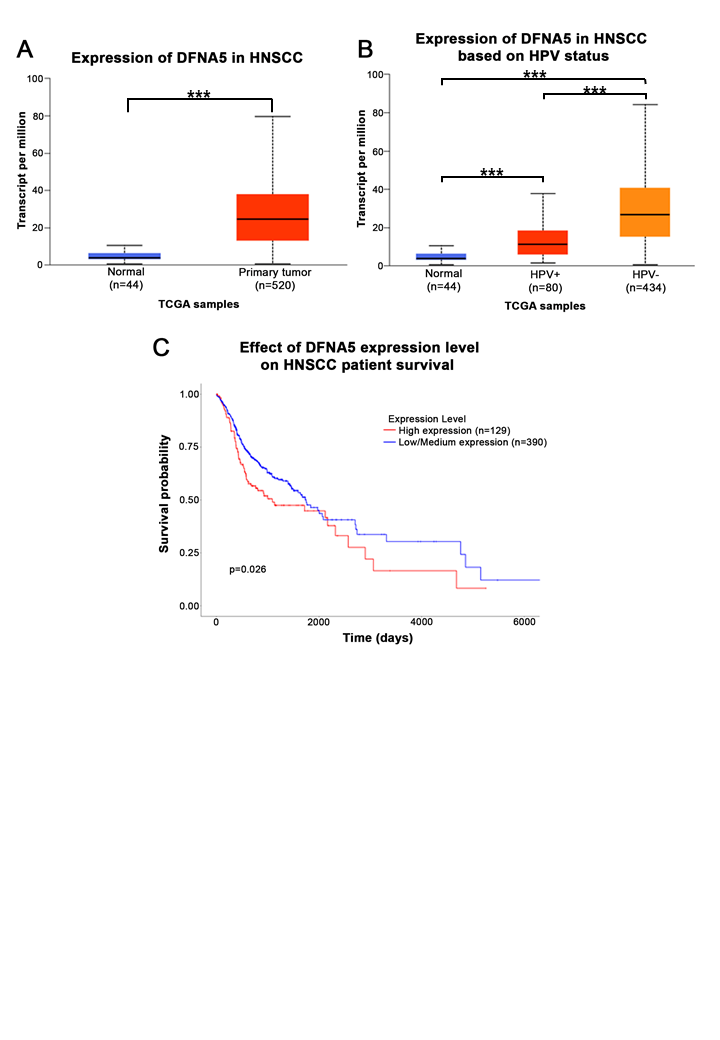
**

**Supplementary figure 7. TCGA analysis of the expression of GSDME in HNSCC patients.** A) Transcriptomic analysis of the expression of GSDME in normal tissue compared to primary tumor samples from HNSCC patients. B) Expression of GSDME in normal tissue compared to primary tumor samples from HPV^+^ and HPV^-^ HNSCC patients. B) Effect of the GSDME expression level on HNSCC patient survival. Analysis was performed using data from the TCGA with the UALCAN analysis software. *** p<0.001.


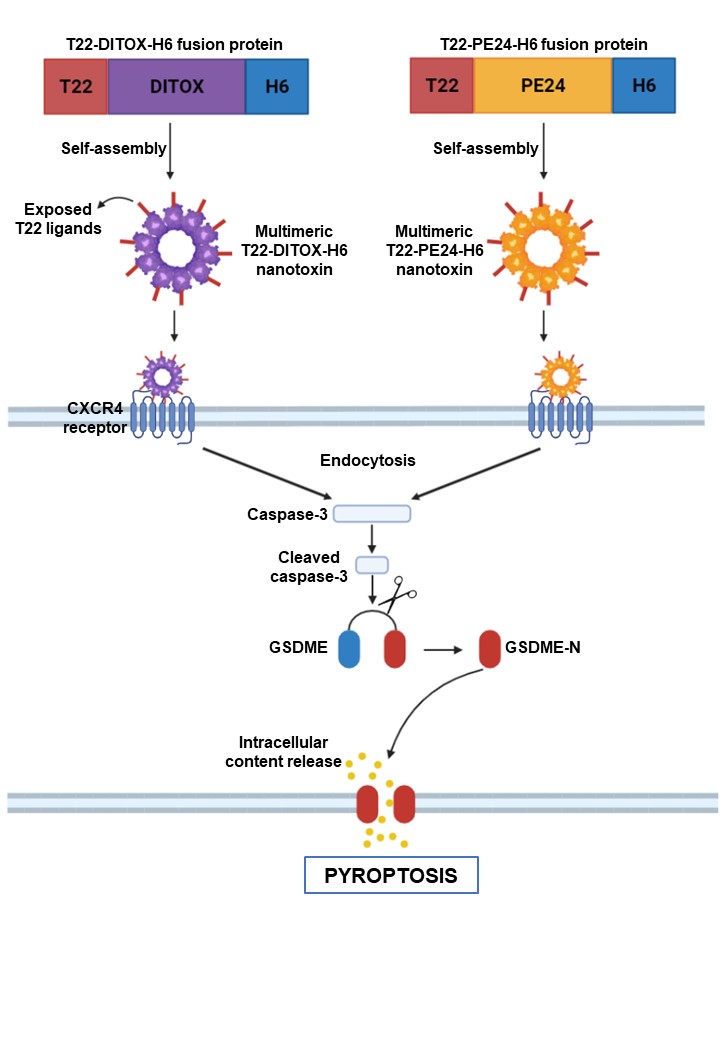


**Supplementary figure 8. Schematic summary of the mechanism of action of T22-PE24-H6 and T22-DITOX-H6 nanotoxins.** Representation of the T22-PE24-H6 and T22-DITOX-H6 fusion proteins and multimeric structures. Interaction of the nanotoxins with the CXCR4 receptor in the cell membrane, leading to their internalization by endocytosis. Toxin domains inside the cell trigger the caspase-3 activation, which in turn induces the cleavage of the GSDME, liberating the GSDME-N terminal domain. Several GSDME-N domains oligomerize to form pores in the cell membrane, leading to the release of the intracellular content and the activation of pyroptosis. Created using BioRender.


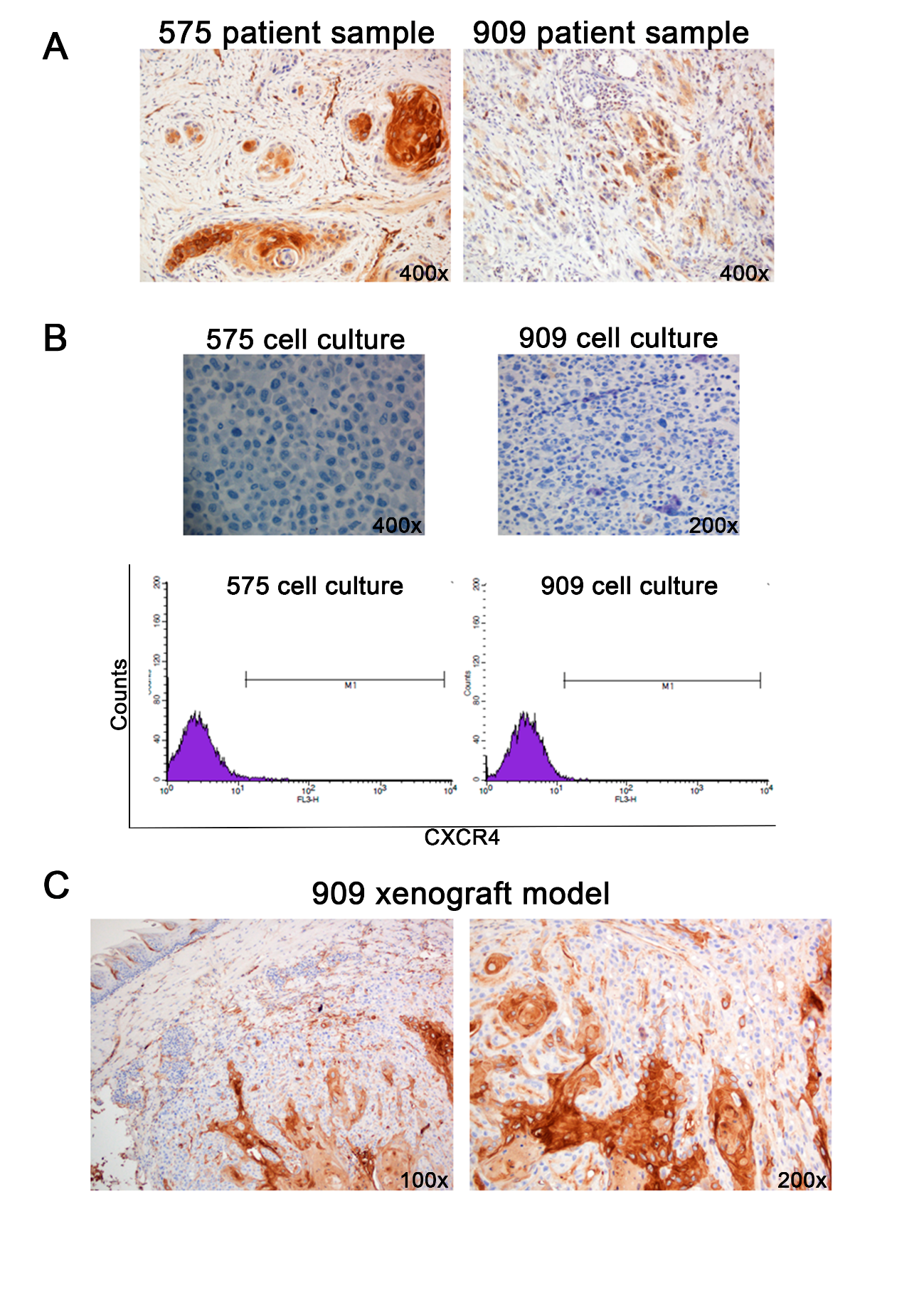


**Supplementary figure 9. CXCR4 expression in 575 and 909 patient-derived cell cultures.** A) CXCR4 expression in the original patient samples for 575 and 909 by immunohistochemistry. B) Immunocytochemical and flow cytometry analysis of 575 and 909 patient samples cultured in vitro. C) Immunohistochemical analysis of CXCR4 expression in tumors generated from 909 cell cultures implanted orthotopically in immunodeficient mice.
